# STAT3 activity is necessary and sufficient for the development of immune-mediated myocarditis in mice and promotes progression to dilated cardiomyopathy

**DOI:** 10.1002/emmm.201201876

**Published:** 2013-03-05

**Authors:** Annalisa Camporeale, Francesca Marino, Anna Papageorgiou, Paolo Carai, Sara Fornero, Steven Fletcher, Brent D G Page, Patrick Gunning, Marco Forni, Roberto Chiarle, Mara Morello, Ole Jensen, Renzo Levi, Stephane Heymans, Valeria Poli

**Affiliations:** 1 Department of Biotechnology and Life Sciences, Molecular Biotechnology Center, University of TorinoTorino, Italy; 2 Department of Cardiology, Center for Heart Failure Research, CARIM, Maastricht UniversityThe Netherlands; 3 Center for Molecular and Cardiovascular Biology (CMVB), University of LeuvenBelgium; 4 Netherlands Heart Institute (ICIN)Utrecht, The Netherlands; 5 Department of Life Sciences and Systems Biology, University of TorinoTorino, Italy; 6 Department of Chemistry, University of TorontoMississauga Road North, Mississauga, ON, Canada; 7 Department of Biomedical Sciences and Human Oncology and CERMS, University of TorinoTorino, Italy; 8 Department of Medical Science, University of TorinoTorino, Italy; 9 Department of Biochemistry and Molecular Biology, University of Southern DenmarkOdense M, Denmark

**Keywords:** complement C3, experimental autoimmune myocarditis, interleukin-6, immune-mediated myocarditis, STAT3

## Abstract

Myocarditis, often triggered by viral infection, may lead to heart auto-immunity and dilated cardiomyopathy. What determines the switch between disease resolution and progression is however incompletely understood. We show that pharmacological inhibition of STAT3, the main mediator of IL-6 signalling and of Th17-cell differentiation, protects mice from the development of Experimental Auto-immune Myocarditis reducing liver production of the complement component C3, and can act therapeutically when administered at disease peak. Further, we demonstrate that STAT3 is sufficient when constitutively active for triggering the onset of immune-mediated myocarditis, involving enhanced complement C3 production and IL-6 signalling amplification in the liver. Disease development can be prevented by C3 depletion and IL-6 receptor neutralization. This appears to be relevant to disease pathogenesis in humans, since acute myocarditis patients display significantly elevated circulating IL-6 and C3 levels and activated heart STAT3. Thus, aberrant IL-6/STAT3-mediated induction of liver acute phase response genes including C3, which occurs as a consequence of pre-existing inflammatory conditions, might represent an important factor determining the degree of myocarditis and its clinical outcome.

## INTRODUCTION

The transcription factor signal transducer and activator of transcription (STAT)3 was first isolated as Acute Phase Response Factor, essential for liver transcriptional responses to acute inflammation (Alonzi et al, 2001), and was later shown to be involved in the signalling of a wide variety of cytokines, growth factors and oncogenes (Poli et al, 2003). Depending on its modalities, STAT3 activation can either be beneficial or pathogenic in a number of conditions. For example, cytokine-mediated activation, controlled by tight negative feedback mechanisms, is required for mammary gland involution (Chapman et al, 1999), contributes to stimulate efficient immune reactions (Fornek et al, 2006) and plays a protective role in myocardial infarction and ischemia (Hilfiker-Kleiner et al, 2004, 2007). On the other hand, its constitutive activity is believed to exert pro-oncogenic functions (Demaria et al, 2012; Yu et al, 2009). Knock-in mice where the wild type (WT) STAT3 allele was replaced by its constitutively active artificial mutant form, STAT3C, were recently generated in our laboratory. These mice display low but continuous STAT3 activity in all tissues, which led to accelerated oncogene-mediated breast tumourigenesis and increased metastasis, altered energy metabolism and predisposition to tumour transformation (Barbieri et al, 2010; Demaria et al, 2010, 2012). Aberrant STAT3 activation often occurs downstream of chronic IL-6 production, which gives rise to a feed-forward loop leading to inflammation-driven tumourigenesis (Li et al, 2011). Indeed, both systemic- and locally sustained IL-6 production are considered a hallmark of chronic inflammation, which can in turn also lead to the development of auto-immunity (Camporeale & Poli, 2012). Moreover, IL-6 is required to initiate the differentiation of Th17 cells, a subset of IL-17-producing pro-inflammatory CD4^+^ T helper cells (Veldhoen et al, 2006). Th17-cell differentiation, which is strongly STAT3-dependent (Harris et al, 2007), is in turn implicated in the pathogenesis of several auto-immune diseases (Steinman, 2007). Additionally, both Th17 cells and IL-6 have been shown to participate in the pathogenesis of experimental auto-immune myocarditis (EAM; Eriksson et al, 2003; Yamashita et al, 2011), which can be triggered in the mouse by immunization with α-cardiac myosin peptide (Pummerer et al, 1996).

Myocarditis is an inflammatory heart condition that can be caused by common viral pathogens with a direct myopathic effect such as, for example coxsackievirus B3 virus (CVB3; Rose, 2009), and is characterized by an inflammatory infiltrate composed of abundant myeloid cells and CD4^+^ T lymphocytes, features that are reproduced in the EAM murine model (Baldeviano et al, 2010; Nindl et al, 2012). The infection can trigger a potent T-cell mediated immune response characterized by high titres of IgG auto-antibodies to cardiac α-myosin and other heart proteins (Caforio et al, 2002). Immune-mediated myocarditis can lead to dilated cardiomyopathy (DCM) in about one third of the cases, and represents a frequent cause of cardiac failure particularly in young adults (Schultheiss et al, 2011). IL-17 has recently been shown to play a pathogenic role in post-myocarditis remodelling and DCM development in mice, suggesting that excessive Th17 lymphocytes activity may be one of the factors determining disease progression (Baldeviano et al, 2010; Nindl et al, 2012). Which other factors determine the switch between disease resolution and progression is however not well understood.

The role of STAT3 in cardiac inflammation has been assessed by expressing the negative feedback factor SOCS3 or by deleting STAT3, both specifically in cardiomyocites. Similar to its known beneficial functions during myocardial infarction, physiological activity of STAT3 in the heart plays a protective role in a model of myocarditis mediated by CVB3 infection (Lindner et al, 2012; Yajima et al, 2006). In contrast, enhanced heart-specific STAT3 activity led to increased inflammation upon myocardial infarction (Hilfiker-Kleiner et al, 2010), underlining the importance of a correct control of STAT3 activation.

Here we show that pharmacological inhibition of STAT3, the main mediator of IL-6 signalling and of Th17-cell differentiation, protects mice from the development of EAM and can improve disease outcome when administered after disease development. Further, we demonstrate that STAT3 is sufficient when constitutively active for triggering the onset of auto-immune myocarditis, involving enhanced complement C3 production and IL-6 signalling amplification in the liver. Disease development can be prevented by C3 depletion and IL-6 receptor neutralization. Thus, uncontrolled systemic STAT3 activation appears to represent a crucial factor in the pathogenesis of immune-mediated myocarditis.

## RESULTS

### STAT3 inhibition abrogates the development of EAM

**Figure 1:**
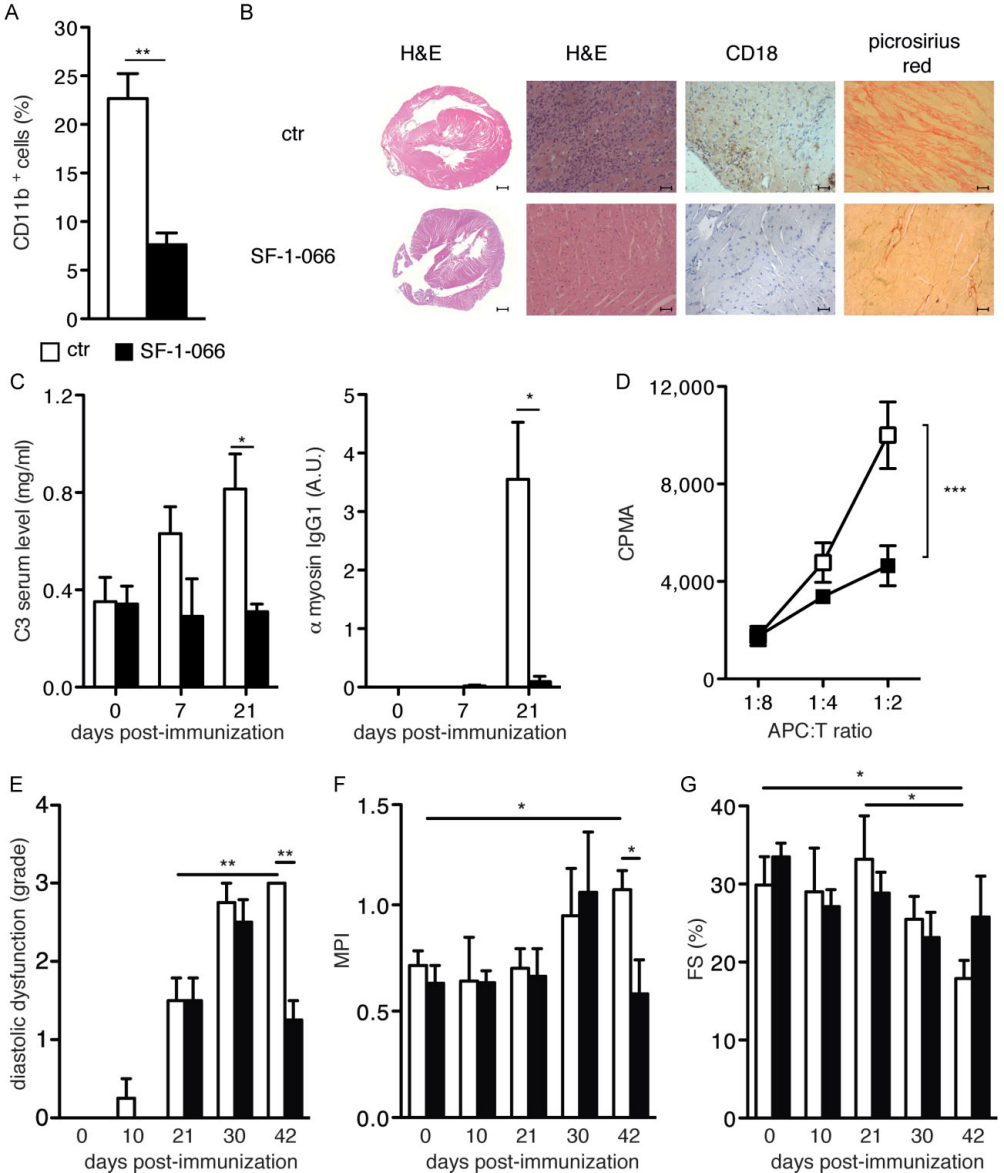
EAM development is dependent on STAT3 activity. Wild type BALB/c mice were immunized with an α-myosin peptide as described in the Materials and Methods section, with or without STAT3 inhibition via i.p. injection of SF-1-066. SF-1-066 was either administered from day 0, and mice sacrificed at day 21 (A–D), or from day 21, and mice sacrificed at day 42 (E–F). Data are plotted as mean ± SEM of three independent experiments. A. Flow cytometry of CD11b^+^ infiltrating cells. The hearts of untreated (white bars, *n* = 11) or SF-1-066-treated mice (black bars, *n* = 8) were analysed. **p = 0.002. B. Analysis of representative heart samples of mice from (A). The indicated stainings were performed. Scale bar: 50 µm, with the exception of the 2× magnification photomicrographs (left panels), where the scale bar is 500 µm. C. C3 ELISA and anti-myosin IgG1. Sera from untreated (white bars, *n* = 5) and SF-1-066-treated (black bars, *n* = 5) mice with EAM at the indicated days after immunization were analysed. C3, *p = 0.027; α-myosin IgG, *p = 0.024. D. T lymphocytes proliferation assay. Thymidine incorporation was measured on splenic CD4^+^ cells isolated from the above mice, upon 3 days of stimulation with APCs pulsed with α-myosin peptide. ***p = 0.0006. E. Echocardiographic analysis was performed on BALB/c mice with EAM, either untreated (white bars, *n* = 4) or treated (black bars, *n* = 4) iv with the SF-1-066 STAT3 inhibitor, starting at day 21 post-immunization. Diastolic dysfunction grade was assessed using pulsed Doppler echocardiography and measuring the left ventricular filling velocity (E/A, ratio of left ventricular filling velocities during early and late diastole), the left ventricular filling pressure (*E*/E′, ratio of transmitral and annular velocities) and the decelerating time (DT) as described in the Methods section. **, untreated mice, day 21 *versus* day 42, = 0.0138; untreated *versus* treated mice, day 42, *p* = 0.0058. F. Myocardial Performance Index (MPI) or Tei index was calculated in the same groups as in *E* from a ratio of time intervals (*a* − *b*/*b*) derived with the pulsed Doppler echocardiography, where ‘*a*’ is the interval time between the end and the start of transmitral flow and ‘*b*’ is the left ventricular ejection time. *, untreated mice, day 0 *versus* day 42, *p* = 0.0191; untreated *versus* treated mice, day 42, *p* = 0.0361. G. Fractional shortening (FS) was measured in the same groups as in *E* using M-mode image of left ventricle and calculated as (LVEDd-LVESd)/LVEDd expressed as a percentage (LVEDd = left ventricular end-diastolic diameter; LVESd = left ventricular end-systolic diameter). *, untreated mice, day 0 *versus* day 42, *p* = 0.0313; untreated mice, day 21 *versus* day 42, *p* = 0.0440.

STAT3 is found aberrantly activated, downstream of IL-6, in a number of chronic inflammatory conditions leading to tumour transformation or auto-immunity (Camporeale & Poli, 2012; Li et al, 2011). To assess the role of STAT3 in the development of immune-mediated myocarditis, we utilized the well-established EAM model, where heart disease is induced by means of immunization with an α-myosin peptide (Pummerer et al, 1996). EAM was induced in BALB/c mice, treated or not with the specific STAT3 inhibitor SF-1-066 (Zhang et al, 2010). STAT3 inhibition was effective (Supporting Information Fig S1A and B) and resulted in protection of mice from myocarditis, as shown by the significantly reduced percentages of heart-infiltrating CD11b^+^ cells at sacrifice (Fig 1A) and by virtually undetectable lymphocyte infiltration as assessed by H&E and anti-CD18 staining (Fig 1B). Particularly significant was the absence of fibrosis, a marker of heart damage, evidenced by the failure of picrosirius red to detect collagen fibrils in the SF-1-066-treated mice (Fig 1B). Up-regulation of the complement component C3, a plasma protein whose main site of production is the liver, is know to play a pathogenic role in EAM (Eriksson et al, 2003; Kaya et al, 2001). STAT3 inhibition completely abrogated both the up-regulation of C3 and the development of anti-myosin antibodies, which reached a peak at day 21 post-immunization in the control mice (Fig 1C). Disease protection correlated with impaired myosin-specific CD4^+^ T lymphocytes responses, as assessed *in vitro* in a myosin-specific proliferation assay (Fig 1D). These data clearly demonstrate that STAT3 plays a crucial role in the pathogenesis of EAM, by both enhancing the activation of CD4^+^ T lymphocytes and up-regulating the production of C3 in the liver.

To assess whether STAT3 inhibition could also act therapeutically by preventing the progression of autoimmune myocarditis to DCM, known to occur after day 21 and to peak at day 42 (Cihakova & Rose, 2008), EAM was induced in BALB/c mice followed by administration of the SF-1-066 inhibitor starting at day 21 post-immunization. All mice developed EAM as assessed by the appearance of anti-myosin antibodies and the up-regulation of circulating C3 levels (Supporting Information Fig S1C and D). Echocardiographic analysis of the left ventricle was periodically performed to assess diastolic and systolic function. STAT3 inhibition could afford a significant improvement of the diastolic function at day 42 post-immunization, when DCM is known to reach its peak (Cihakova & Rose, 2008). While all mice displayed similar levels of restrictive cardiomyopathy at days 21 and 30, assessed as grade of diastolic dysfunction as described in the Methods section, by day 42 dysfunction was significantly reduced in SF-1-066-treated mice (Fig 1E). This correlated with reduced ventricular systolic and diastolic dysfunction, as shown by a lower Myocardial Performance Index (MPI; Fig 1F). MPI failed to increase significantly in STAT3-inhibited mice, and at day 42 was indistinguishable from that observed before disease induction. Moreover, while control mice displayed a significant reduction of the fractional shortening (FS) at day 42, FS failed to significantly decrease in the SF-1-066-treated mice, suggesting a better preservation of contractile function (Fig 1G and Supporting Information Fig S1E). Taken together, these data suggest that STAT3, in addition to play a central role in EAM onset, is also crucial for its progression to heart failure and DCM.

### Aggressive myocarditis in knock-in mice expressing constitutively active STAT3

**Figure 2:**
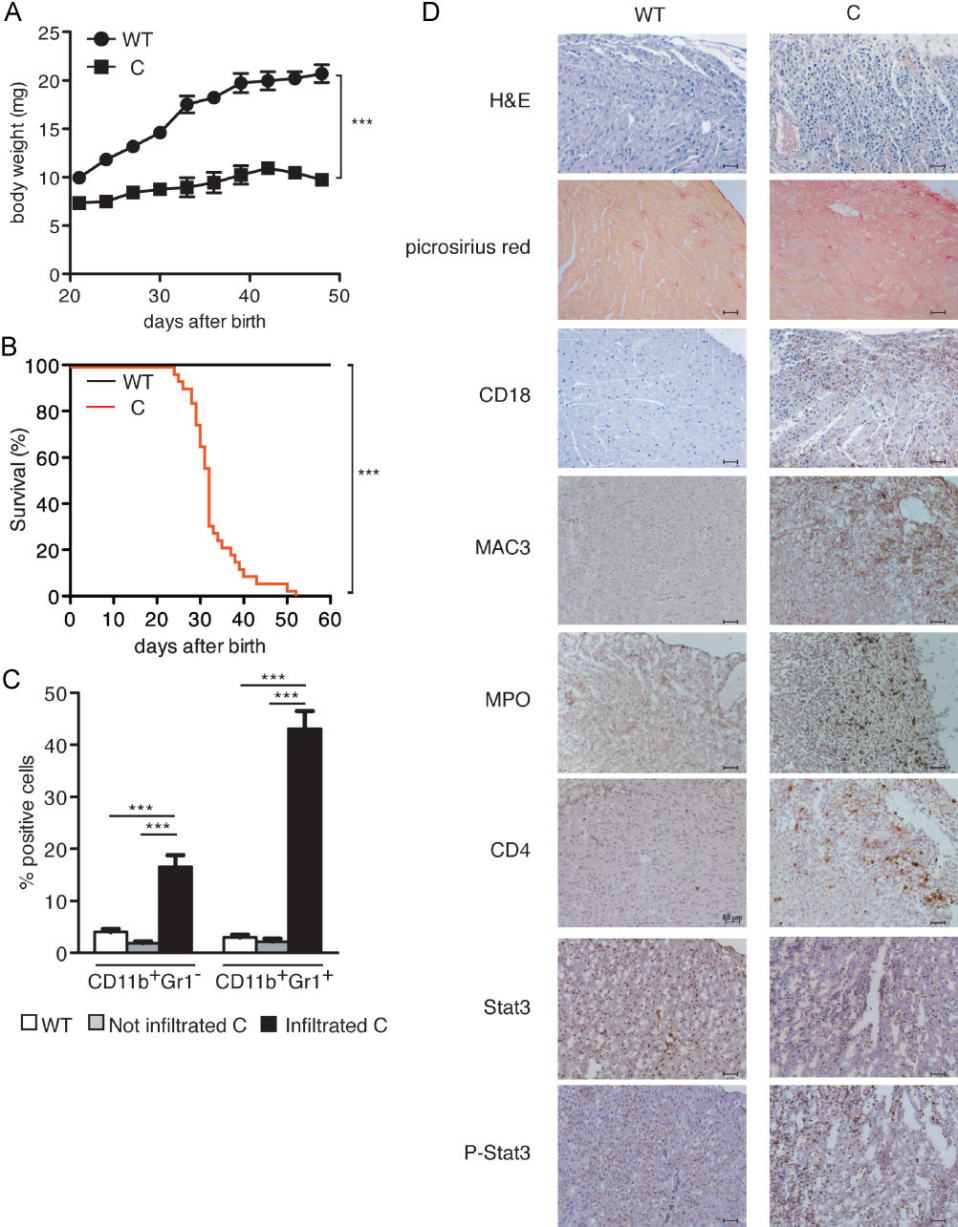
Myocarditis development in Stat3^C/C^ mice. A. Growth curves. 12 Stat3^WT/WT^ (black circles) and Stat3^C/C^ littermates (black squares) were weighed every 3 days starting from the day of birth. ***p < 0.0001. B. Kaplan-Meier survival curves of Stat3^C/C^ (red line) and Stat3^WT/WT^ (black line) mice (*n* = 32). ***p < 0.0001. C. Cytofluorimetric analysis. Data are shown as mean ± SEM of infiltrating CD11b^+^Gr1^−^ and CD11b^+^Gr1^+^ cells in the hearts of Stat3^WT/WT^ (white bars, *n* = 41), non-infiltrated (grey bars, *n* = 17) and infiltrated (black bars, *n* = 29) Stat3^C/C^ mice. ***p < 0.0001. D. Histological, histochemical and immunohistochemical analysis. Hearts from Stat3^WT/WT^ or infiltrated Stat3^C/C^ mice were stained as indicated: haematoxylin and eosin (H&E), picrosirius red, or antibodies against myeloperoxidase (MPO), CD18, CD4, STAT3, P-STAT3. Pictures are representative of at least three mice per group. Scale bar: 50 µm.

**Figure 3:**
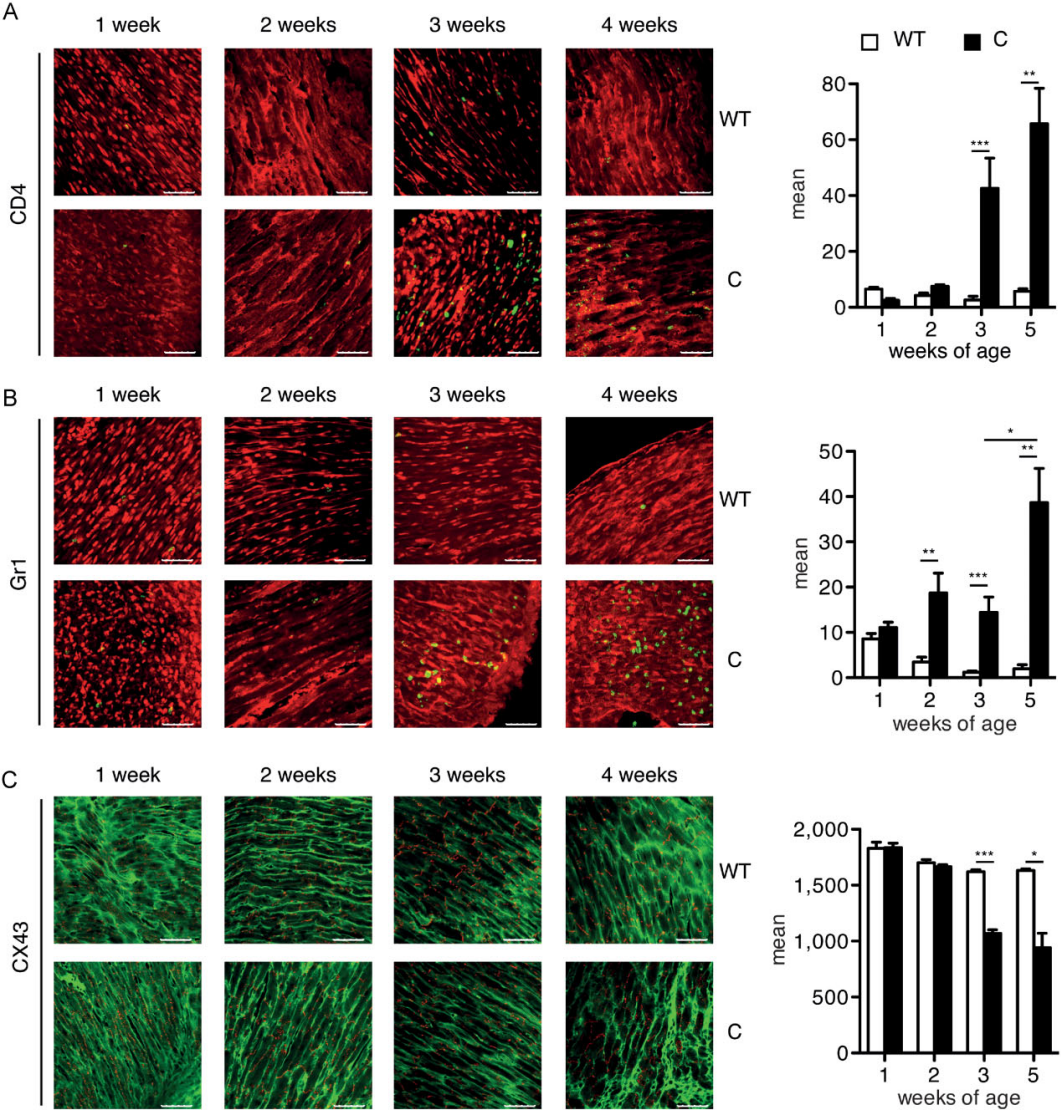
Immunofluorescence analysis of heart infiltration and morphology. Hearts were dissected from Stat3^C/C^ or Stat3^WT/WT^ mice at the indicated weeks of age and stained with the indicated antibodies. Pictures are representative of four to six mice per group. Histograms represent the quantification of fluorescence intensity (mean ± SEM). Scale bar: 50 µm. ***p < 0.001; **p < 0.01; *p < 0.05. A. Anti-CD4, green; anti-alpha actinin, red. B. Anti-Gr1, green; anti-alpha actinin, red. C. Anti-connexin (cx) 43, red; anti-alpha actinin, green.

Knock-in Stat3^C/C^ mice, expressing the constitutively active mutant form STAT3C (Barbieri et al, 2010), were born with mendelian ratios and displayed slightly reduced body weight at birth and blunted growth curves when compared to WT littermates, followed by weight loss and death between 4 and 6 weeks of age (Fig 2A and B). In contrast, heterozygous Stat3^C/WT^ mice displayed normal growth rates and life span. Post-mortem examination revealed massive leukocyte infiltration in the heart, confirmed by immunohistochemical (IHC) staining with the pan-leukocyte marker CD18 (Fig 2D). Almost 60% of the infiltrating population was composed of CD11b^+^ myeloid cells, mostly positive for the granulocyte marker Gr1 (Fig 2C). We established an arbitrary threshold of 10% increase in CD11b^+^ cells in the heart of Stat3^C/C^ *versus* Stat3^WT/WT^ mice in order to define two groups, that is infiltrated/sick *versus* non-infiltrated/healthy Stat3^C/C^ mice. CD11b^+^/Gr1^+^ cells were also significantly expanded in the bone marrow (BM) and the peripheral blood of both infiltrated (i.) and non-infiltrated (n.i.) Stat3^C/C^ mice (BM, 56.1 ± 5.7% n.i., 51.8 ± 3.9% i., compared to 33.8 ± 2.1% in the Stat3^WT/WT^ mice; blood, 54.3 ± 4.1% n.i., 65.0 ± 4.4% i., *versus* 22.9 ± 2.6 in the WT controls). Myeloperoxidase-positive granulocytes, CD4^+^ lymphocytes and MAC3^+^ macrophages were detected by IHC in infiltrated heart sections, together with a strong increase of tyrosine-phosphorylated STAT3 (Fig 2D), and of pro-inflammatory cytokine and chemokine mRNAs in the heart (Supporting Information Fig S2). Heart infiltration correlated with cardiac damage, as shown by the marked reparative fibrosis evidenced by picrosirius red staining of collagen fibrils (Fig 2D). Immunofluorescence (IF) analysis showed increasing numbers of Gr1^+^ cells in Stat3^C/C^ mice already starting from week 2 of age, followed by CD4^+^ T-cell accumulation, detectable from week 3 (Fig 3A and B). Of note, it was only possible to detect small foci of leukocyte infiltration in other organs such as the liver, the spleen and the lymphnodes, but not the skeletal muscle, showing that the dramatic inflammation observed is specific to heart striated muscle and ruling out the involvement of a systemic inflammatory disease (Supporting Information Table S1).

Additionally we analysed connexin 43 (cx43), the major component of gap junctions in the adult heart, whose decrease or delocalization is an established marker of cardiac suffering and heart failure (Fernandez-Cobo et al, 1999; Jansen et al, 2012). The cx43 signal, whilst initially comparable with WT mice, decreased in the hearts of Stat3^C/C^ mice from 3 weeks of age becoming progressively dispersed (Fig 3C), suggesting that heart tissue organization, initially normal, becomes disorganized as a consequence of inflammation, detected at 2 weeks of age with infiltrating Gr1^+^ cells (Fig 3B). This was also confirmed by the irregular α-actinin staining that reveals progressive degradation of cell shape, particularly around the more heavily infiltrated regions (Fig. 3). The described heart damage in the Stat3^C/C^ mice clearly represents a phenotype of ongoing myocarditis, starting to lead to altered heart functionality as judged by echocardiography, since fractional shortening appeared to be significantly reduced (Supporting Information Table S2).

### Myocarditis development in Stat3^C/C^ mice correlates with heart auto-immunity

**Figure 4:**
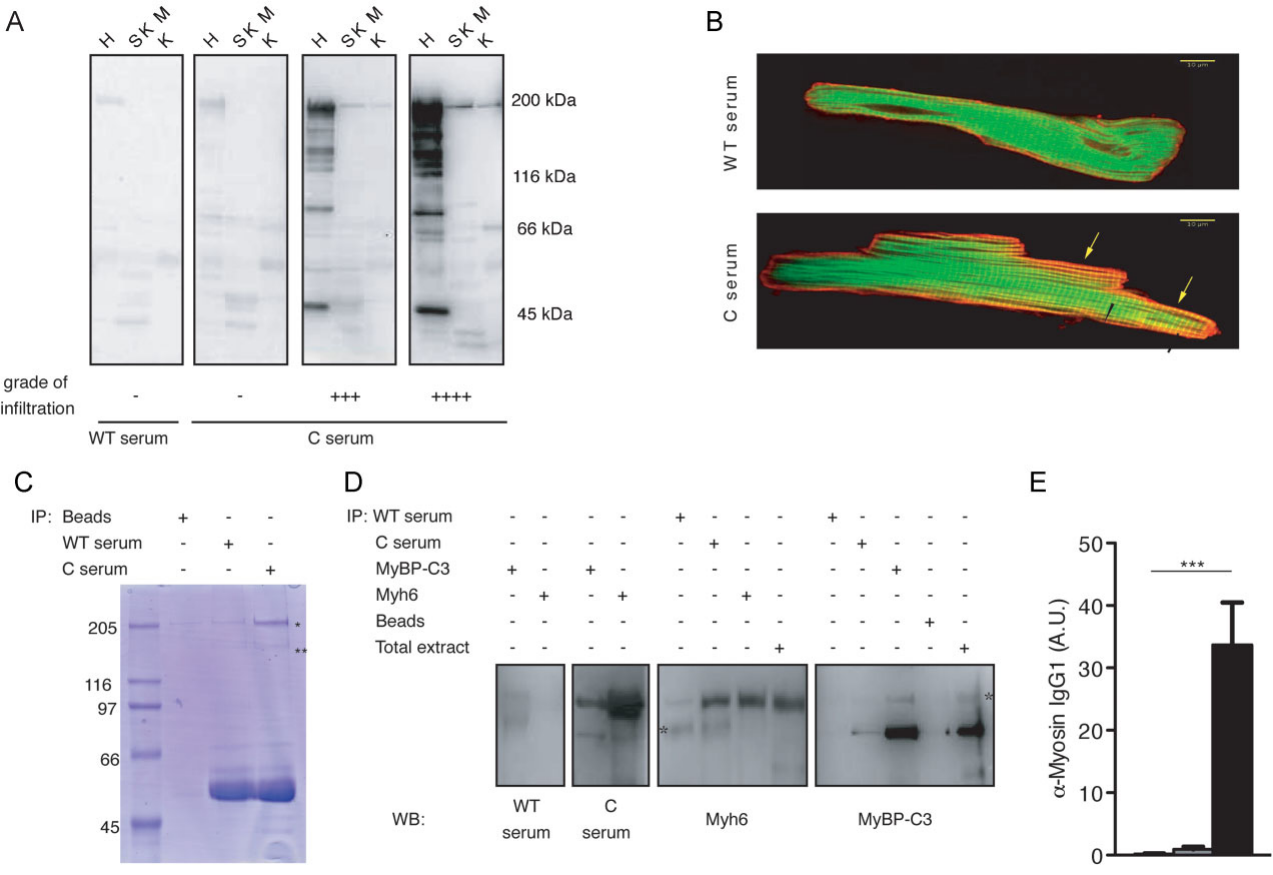
Heart auto-immunity in infiltrated Stat3^C/C^ mice. A. Immunoblot analysis of total protein extracts from heart (H), skeletal muscle (SKM) or kidney (K) with sera from individual Stat3^WT/WT^ (WT) and Stat3^C/C^ (C) mice. The degree of infiltration is shown. B. IF analysis of rat cardiocytes with sera from Stat3^WT/WT^ and Stat3^C/C^ mice. Red: serum decorated with anti-mouse fluorescent antibody; green: phalloidin. Note the strong red staining obtained with Stat3^C/C^ serum, marking a sarcomeric-organized component that partially superimposes with the green phalloidin labelling of actin, resulting in a repetition of yellow bands (arrows), absent in the cell labelled with Stat3^WT/WT^ serum. C. Preparative gel for mass spectrometry. Whole heart extracts were immunoprecipitated with pooled sera and fractionated on SDS–PAGE followed by Coomassie staining. Bands indicated by asterisks were excised and analysed by Mass Spectrometry. D. Whole heart extracts were immunoprecipitated followed by immunoblotting with the indicated sera/antibodies. Data are representative of two to three experiments with individual sera. The asterisks indicate non-specific signals. E. Myosin-specific IgG1 ELISA. Sera from Stat3^WT/WT^ (white bars, *n* = 28), non-infiltrated (grey bars, *n* = 22) and infiltrated (black bars, *n* = 20) Stat3^C/C^ mice were used. Bars represent mean ± SEM of values expressed as arbitrary units (A.U.). ***p < 0.0001.

Western blot analysis of whole heart extracts and IF staining of isolated adult myocytes with sera derived from diseased Stat3^C/C^ mice showed strong reactivity against proteins of the heart but not of other tissues including skeletal muscle, suggesting auto-immunity against heart components (Fig 4A and B). Notably, there was a good correlation between sera reactivity and degree of infiltration, whilst sera from non-infiltrated Stat3^C/C^ and WT mice were equivalent. Mass spectrometry analysis of cardiac extracts immunoprecipitated with pooled sera from Stat3^C/C^ mice identified myosin alpha heavy chain (Myh6), the major component of adult heart myosin, and myosin binding protein C3 (MyBP-C3) as the main recognized antigens (Fig 4C). Results were confirmed using specific anti-Myh6 and anti-MyBP-C3 antibodies (Fig 4D). Anti-myosin alpha chain antibody titres correlated well with heart infiltration, as indicated by ELISA detection of α-myosin-specific IgG1s (Fig 4E). This assay was then routinely used to follow disease progression prior to post-mortem examination.

### Both haematopoietic and non-haematopoietic cells contribute to disease development

**Figure 5:**
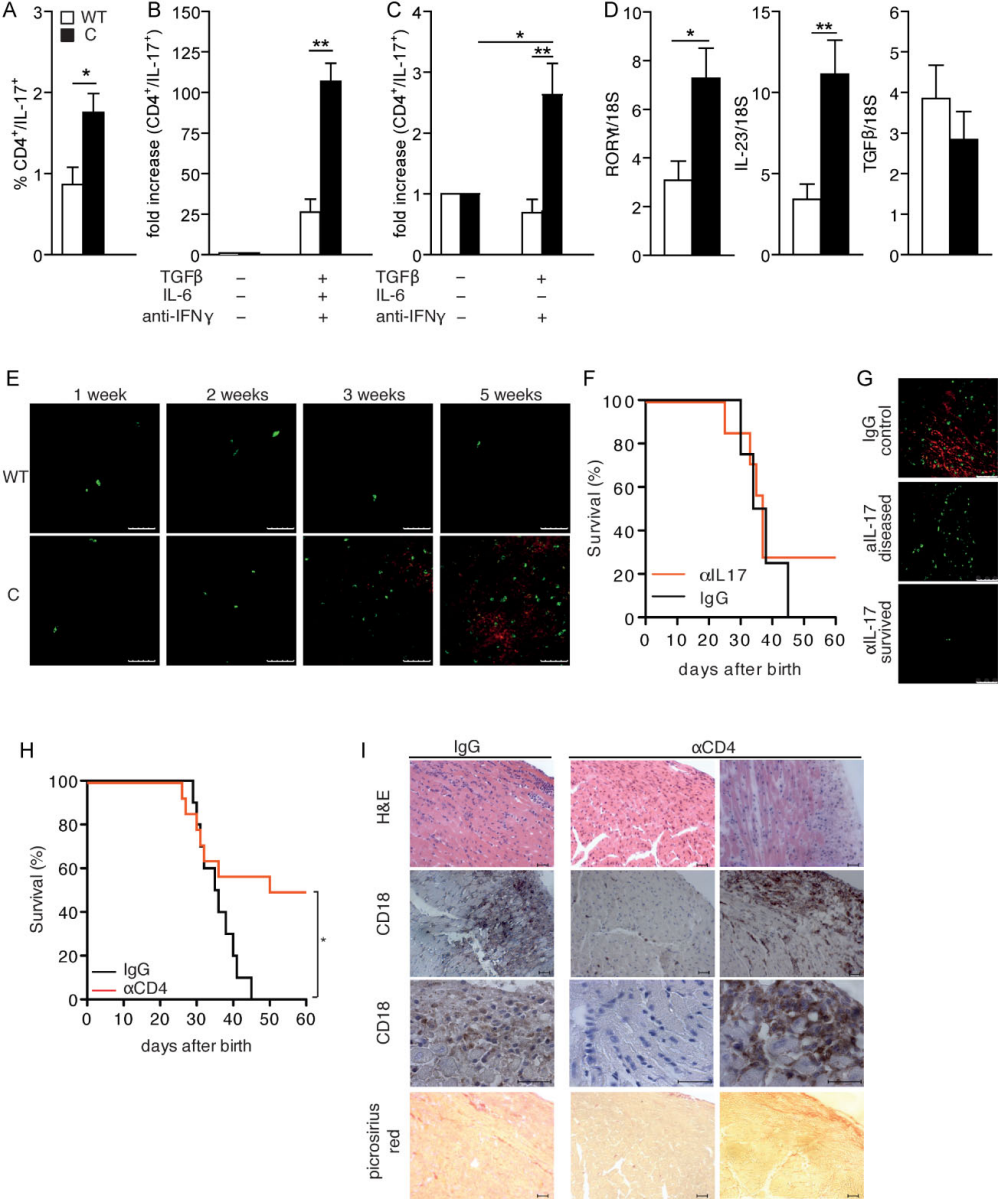
Stat3^C/C^ mice are more prone to Th17 differentiation. A. Cytofluorimetric analysis of IL-17 producing CD4^+^ T cells in the spleen of Stat3^WT/WT^ (white bars, *n* = 6) and Stat3^C/C^ (black bars, *n* = 6) mice. Data are shown as mean ± SEM. *p = 0.015. B. *In vitro* differentiation of Th17 cells. Naïve CD4^+^ cells were purified from spleens of Stat3^WT/WT^ (white bars, *n* = 6) and Stat3^C/C^ (black bars, *n* = 10) mice and treated or not with TGF-β, IL-6 and anti-IFN-γ, followed by cytofluorimetric analysis for CD4 and IL-17. Data are shown as mean ± SEM. **p = 0.001. C. *In vitro* differentiation of Th17 cells without IL-6. Naïve CD4^+^ cells were purified from spleens of Stat3^WT/WT^ (white bars, *n* = 6) and Stat3^C/C^ (black bars, *n* = 6) mice, treated or not with TGF-β and anti-IFN-γ and analysed as above. Note the different scales between (B) and (C). Data are shown as mean ± SEM. **p = 0.006 (Stat3^WT/WT^ *versus* Stat3^C/C^ cells); *p = 0.025 (untreated *versus* treated Stat3^C/C^ cells). D. Taqman RT-PCR analysis on total RNA from the spleen of Stat3^WT/WT^ (white bars, *n* = 7) and Stat3^C/C^ (black bars, *n* = 7) mice. Data are shown as mean ± SEM. *p = 0.018 (RORγt); **p = 0.0057 (IL-23). E. IL-17 (red) and CD4 (green) IF staining of hearts from Stat3^WT/WT^ (WT) and Stat3^C/C^ (C) mice at the indicated weeks of age. Representative samples of three mice per group are shown. Scale bar: 50 µm. F. Kaplan-Meier survival curves of Stat3^C/C^ mice treated with anti-IL-17 neutralizing mAbs (red line, *n* = 7) or with total IgG as controls (black line, *n* = 4). G. IL-17 (red) and CD4 (green) IF staining of hearts from anti IL-17-treated and control mice as above. Representative samples from survived (*n* = 2) or diseased (*n* = 5) mice are shown. Scale bar: 50 µm. H. Kaplan-Meier survival curves of Stat3^C/C^ mice treated with anti-CD4 neutralizing mAbs (red line, *n* = 13) or with total IgGs as controls (black line, *n* = 10). *p = 0.026. I. The hearts of mice from (H) were analysed as indicated. IgG, control mice; for the aCD4-treated mice, samples from survived mice are shown on the left, and those from dead mice on the right. Scale bar: 50 µm.

CD4^+^ Th17 lymphocytes are known to play a role in EAM pathogenesis (Rangachari et al, 2006; Sonderegger et al, 2006) and to require STAT3 for their differentiation (Harris et al, 2007; Yang et al, 2007). Th17 cells were significantly more abundant in the spleen of Stat3^C/C^ mice compared to their WT counterparts (Fig 5A), and naïve spleen-derived CD4^+^ cells from Stat3^C/C^ mice exhibited a threefold enhanced ability to differentiate towards the Th17 lineage as compared to controls (Fig 5B). Further, when treated with TGF-β in the absence of IL-6, only CD4^+^ Stat3^C/C^ cells could differentiate, albeit at low efficiency, into IL-17-producing cells, suggesting that STAT3 constitutive activity can partly override the need for IL-6 in Th17 differentiation (Fig 5C). In agreement with the expanded Th17 population, the mRNAs for RORγT and IL-23 were significantly elevated in the spleen of Stat3^C/C^ mice (Fig 5D). Accordingly IL-17, already displaying an increasing trend at the mRNA level in the heart (Supporting Information Fig S2), was detected by IF in the hearts of Stat3^C/C^ mice from 3 weeks of age (Fig 5E), correlating with the appearance of CD4^+^ cells and increasing with disease progression. Thus, constitutively active STAT3 enhances Th17 function, which in turn might contribute to exacerbate myocarditis by maintaining inflammation and increasing tissue damage. To assess the importance of IL-17 in our system, we treated Stat3^C/C^ mice with neutralizing anti-IL-17A antibodies or with control IgGs. Only two out of seven treated mice (25%) displayed increased survival (up to 60 days of age, Fig 5F). IL-17 neutralization only moderately decreased leukocyte infiltration and fibrosis in surviving, but not in succumbing, mice (Supporting Information Fig S3A). Anti-myosin antibody levels and heart infiltration by CD4^+^ cells were significantly decreased in the surviving mice, but comparable to the untreated mice in the succumbing animals (Supporting Information Fig S3B and Fig 5G). These observations suggest a limited role for Th17 cells in our model. In contrast, CD4 depletion – by means of neutralizing anti-CD4 antibodies – could rescue 50% of the Stat3^C/C^ mice from death up to 60 days of age, when they were sacrificed for analysis (Fig 5H and Supporting Information Fig S3C–E). Surviving anti-CD4-treated mice developed a significantly milder disease, with strongly reduced heart infiltration, tissue damage and auto-antibody levels (Fig 5I and Supporting Information Fig S3C and D). The discrepancy between the results obtained with IL-17 neutralization and CD4 depletion may be explained by a differential efficiency of the two treatments. Additionally, other CD4^+^ cell populations may be involved in myocarditis development and outcome. Indeed, Th1 cells percentages and *in vitro* differentiation potential are preserved in the Stat3^C/C^ mice (Supporting Information Fig S3F and G), and the number of spleen-derived Th2 cells is significantly increased (Supporting Information Fig S3F). This may be a consequence of *in vivo* Th17 cells expansion (Stritesky et al, 2011), as the *in vitro* Th2 differentiation potential is instead normal (Supporting Information Fig S3H).

**Figure 6:**
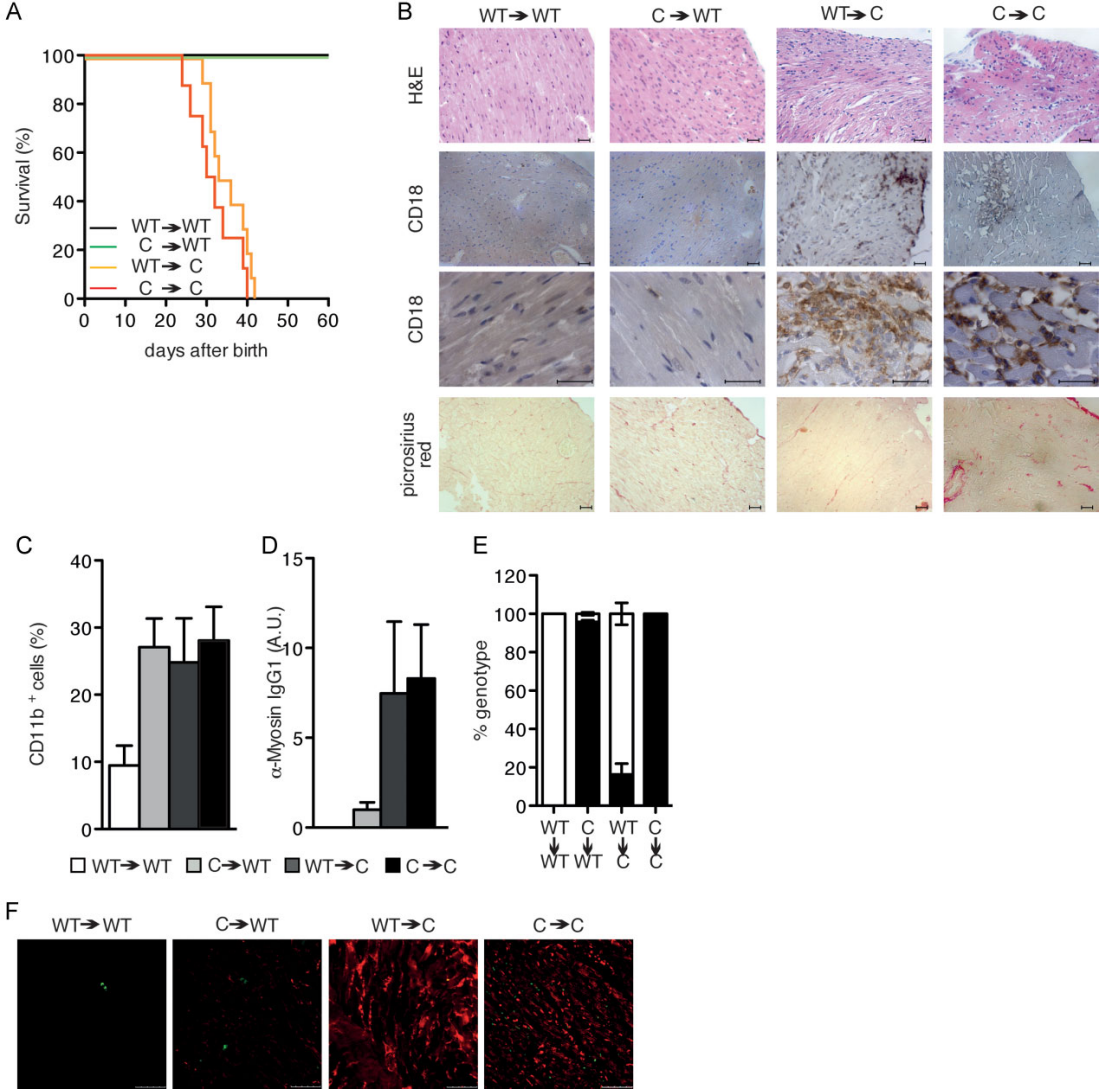
Both haematopoietic and non-haematopoietic compartments are involved in myocarditis development. A. Kaplan-Meier survival curves showing survival of bone marrow chimeras. Control Stat3^WT/WT^-> Stat3^WT/WT^, black line, *n* = 14; Stat3^C/C^->Stat3^WT/WT^, green line, *n* = 15; Stat3^WT/WT^->Stat3^C/C^, orange line, *n* = 9; and control Stat3^C/C^->Stat3^C/C^, red line, *n* = 6. B. Representative heart histochemical analysis of mice from (A). Scale bar: 50 µm. C. Heart-infiltrating CD11b^+^ cells measured by flow cytometry in mice from (A), shown as mean ± SEM of percentages of CD11b^+^ cells. Stat3^WT/WT^->Stat3^WT/WT^ (white bars, *n* = 14); Stat3^C/C^->Stat3^WT/WT^ (grey bars, *n* = 15); Stat3^WT/WT^->Stat3^C/C^ (dark grey bars, *n* = 10) and Stat3^C/C^->Stat3^C/C^ (black bars, *n* = 4). D. Myosin-specific serum IgG1 levels measured by ELISA in mice from A, shown as mean ± SEM of values expressed as arbitrary units. Bars as in (C), numbers as in (A). E. The relative abundance of the STAT3WT (white bar) and STAT3C (black bar) alleles was measured by SYBR Green real-time PCR in the blood of transplanted mice at the time of sacrifice. F. IF analysis of C3 deposition in the hearts of mice from (C). Pictures are representative of six mice per group. Scale bar: 50 µm.

Since the rescue obtained by CD4 depletion was only partial and the STAT3C allele is expressed also in non-haematopoietic cells, we decided to assess the relative contribution to disease development of haematopoietic *versus* non-haematopoietic cells via the generation of BM chimeras. First, lethally irradiated Stat3^WT/WT^ mice were reconstituted with Stat3^C/C^ BM cells (C->WT). These mice developed mild myocarditis, surviving for up to 60 days of age (Fig 6A). Heart infiltration by CD18^+^ cells, fibrosis and circulating auto-antibody levels were reduced with respect to controls, that is Stat3^C/C^ mice reconstituted with Stat3^C/C^ BM cells (C->C), whilst the percentage of infiltrating CD11b^+^ cells was similar (Fig 6B–D). Interestingly, reconstitution of Stat3^C/C^ mice with Stat3^WT/WT^ haematopoietic cells (C->WT) resulted in the development of a more aggressive myocarditis than that observed in the C->WT transplanted mice. Heart infiltration by CD18^+^ cells and auto-antibody levels were similarly high in WT->C and C->C mice, while fibrosis was reduced (Fig 6B and D). The percentage of infiltrating CD11b^+^ cells was similar in the three groups as compared to the control WT->WT chimeras (Fig 6C). Despite similar death rates and age, the histopathological alteration observed in the WT->C and the C->C groups was reduced with respect to non-transplanted Stat3^C/C^ mice. This may be due to the early death after the transfer (between 12 and 15 days) that limited the accumulation and pathogenic action of the transplanted haematopoietic cells. These data clearly show that both haematopoietic and non-haematopoietic cells participate to the pathogenesis of myocarditis in the Stat3^C/C^ mice. Additionally, we observed that heart deposition of complement component C3 was different in the different groups of chimeras (Fig 6F). Interestingly, while C3 was almost undetectable in both groups of recipient WT mice, we could detect comparably abundant C3 deposition in recipient Stat3^C/C^ mice transplanted with either WT or Stat3^C/C^ BM cells, correlating with their failure to survive. This observation suggests that C3 production, contributed by non-haematopoietic Stat3^C/C^ cells, and its accumulation in the heart, may represent a crucial pathogenic factor in our model.

### Myocarditis is dependent on high C3 production

**Figure 7:**
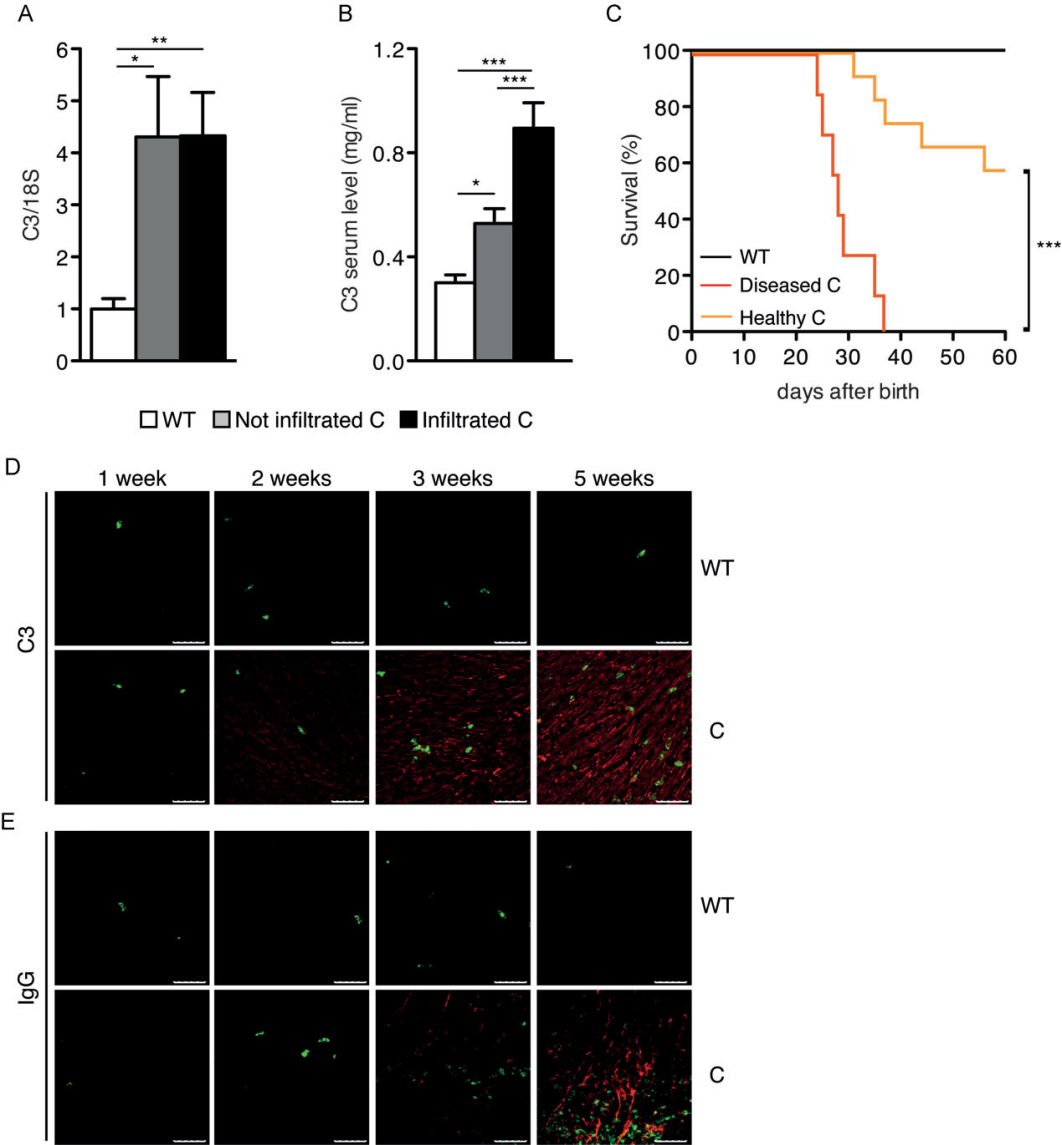
Pathogenic role of C3. A. C3 protein levels measured by ELISA in the serum from Stat3^WT/WT^ (white bars, *n* = 26), non-infiltrated (grey bars, *n* = 26) and infiltrated (black bars, *n* = 28) Stat3^C/C^ mice. Data are shown as mean ± SEM. *p = 0.034; ***p < 0.0001. B. C3 mRNA levels measured by Taqman RT-PCR on liver RNA from the same mice as in (A). Data are shown as mean ± SEM. *p = 0.013 (Stat3^WT/WT^ *vs* non-infiltrated Stat3^C/C^); **p = 0.0086 (Stat3^WT/WT^ *vs* infiltrated Stat3^C/C^). C. Survival curves of mice after *in vivo* complement depletion. CVF-treated Stat3^WT/WT^ (black line, *n* = 8), non-infiltrated (orange line, *n* = 7) and infiltrated (red line, *n* = 12) Stat3^C/C^ mice were analysed. ***p < 0.0001. D. IF analysis of C3 localization in the hearts of Stat3^C/C^ and Stat3^WT/WT^ mice at the indicated ages in weeks. Green: CD4; Red: C3. Pictures are representative of six mice per genotype. Scale bar: 50 µm. E. Immunofluorescence analysis of IgG deposition (in red) in the same samples from (D). Scale bar: 50 µm.

The above observation prompted us to examine the role of C3 in the pathogenesis of myocarditis in the Stat3^C/C^ mice. Indeed, C3 belongs to those serum proteins whose transcription is induced in the liver during the acute phase of inflammation, via IL-6-dependent STAT3 activation (Alonzi et al, 2001), and its pathogenic role is particularly relevant downstream of IL-6 during the early, inductive phases of EAM (Eriksson et al, 2003; Kaya et al, 2001). Interestingly, C3 mRNA levels were significantly elevated in the liver of Stat3^C/C^ mice compared to their WT littermate controls even prior to disease development, correlating with significantly increased abundance of the circulating protein (Fig 7A and B). To assess the potential pathogenic role of C3 in our model, mice were treated with cobra venom factor (CVF) to achieve complement depletion (Kaya et al, 2001). This did not affect the onset or severity of myocarditis in those Stat3^C/C^ mice already showing high C3 or anti-α-myosin antibodies at the time of treatment, in agreement with the predicted role of C3 in the inductive phase (Supporting Information Fig S4A and B and Fig 7C). In contrast, almost 60% of the mice not yet displaying high C3 or auto-antibody levels were rescued by complement depletion (Supporting Information Fig S4A and B and Fig 7C), showing significantly reduced auto-antibody titres, heart infiltration and fibrosis (Supporting Information Fig S4C–E). Thus, STAT3-dependent early C3 up-regulation may indeed represent a disease-initiating factor occurring in the liver, as also supported by the observation that neither IL-17A neutralization nor CD4 depletion could reduce C3 circulating levels (Supporting Information Fig S5A and B).

Accordingly, C3 could be detected by IF and IHC in the cardiac tissue of Stat3^C/C^ mice at just 2 weeks of age, correlating with the increase of Gr1^+^ cells and before the detection of CD4^+^ cells and the appearance of anti-myosin antibodies (Fig 7D and Supporting Information Fig S4F). Heart C3 deposition progressively increased with disease development, followed by IgG deposition starting from 3 weeks of age, time of detection of the first CD4^+^ T lymphocytes infiltrating the heart (Fig 7D and E and Supporting Information Fig S4F).

### IL-6 receptor neutralization can significantly rescue myocarditis development

**Figure 8:**
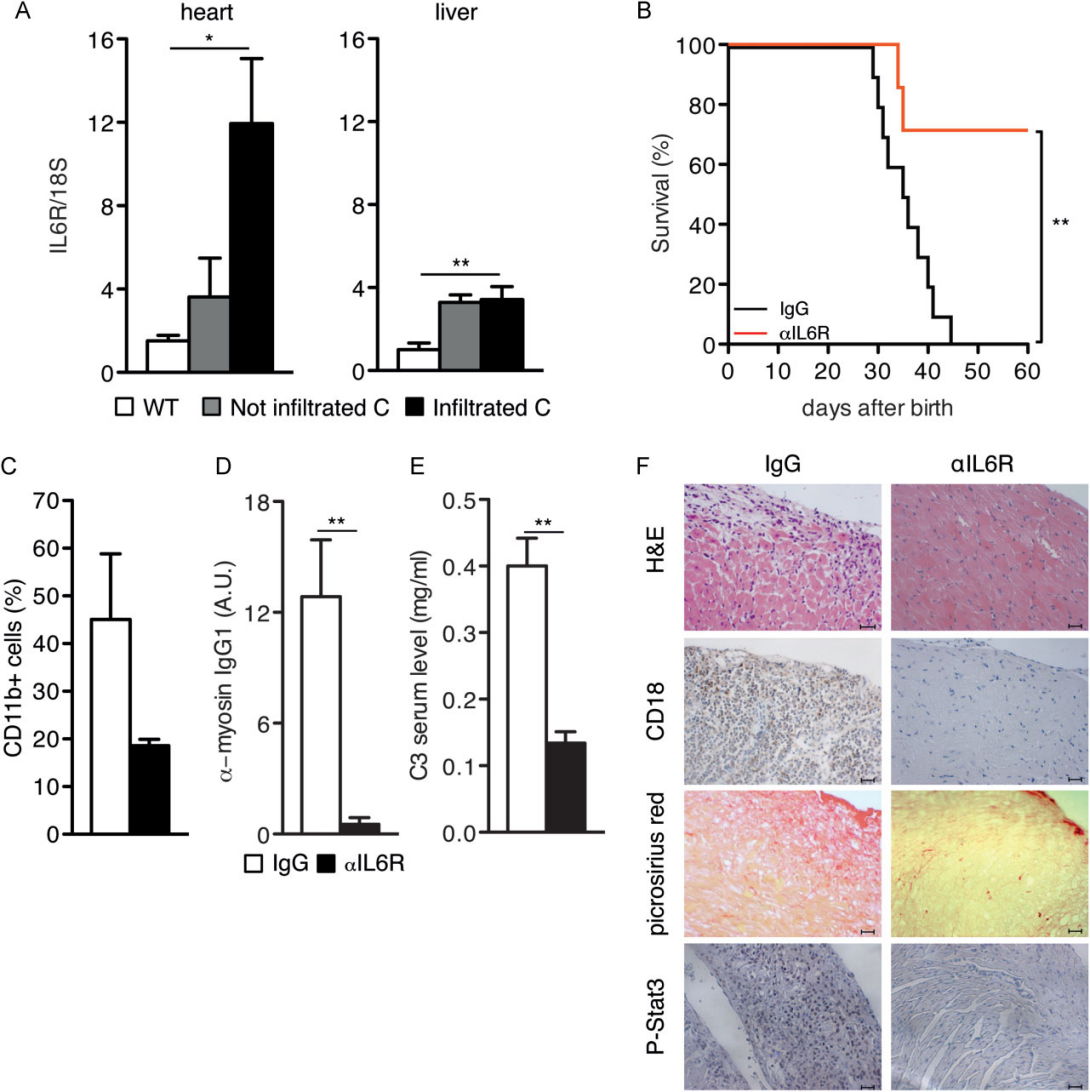
Pathogenic role of IL-6. A. IL-6Ra mRNA levels (mean ± SEM of normalized values) were measured by Taqman RT-PCR in the hearts or livers of Stat3^WT/WT^ (white bars, *n* = 10), non-infiltrated (grey bars, *n* = 9) and infiltrated (black bars, *n* = 8) Stat3^C/C^ mice. * heart, Stat3^WT/WT^ *versus* non-infiltrated Stat3^C/C^, *p* = 0.034; heart, Stat3^WT/WT^ *versus* infiltrated Stat3^C/C^, *p* = 0.008; ** liver, Stat3^WT/WT^ *versus* non-infiltrated Stat3^C/C^, *p* = 0.002; liver, Stat3^WT/WT^ *versus* infiltrated Stat3^C/C^, *p* = 0.001. B. Kaplan-Meier survival curves of Stat3^C/C^ mice treated with anti-IL-6R-neutralizing antibodies (red line, *n* = 7) or with total IgGs (black line, *n* = 10) as control, starting from 2 weeks of age. **p = 0.0058. C. Flow cytometry of CD11b^+^ heart-infiltrating cells in mice from (B). Results are shown as mean ± SEM of the percentage of positive cells. IgG-treated (white bars, *n* = 5); surviving anti-IL6R-treated (black bars, *n* = 3). D. Anti-myosin IgG1 ELISA in mice from (B). Results are shown as mean ± SEM of signal intensity in arbitrary units (A.U.) in the sera from IgG-treated (white bars, *n* = 10) and surviving anti-IL6R-treated (black bars, *n* = *5*) Stat3^C/C^ mice, sacrificed at 60 days of age. **p = 0.0073. E. Circulating C3 ELISA. The same sera as in (D) were analysed. **p = 0.0011. F. Representative heart histochemical analysis of mice from (C). Scale bar: 50 µm.

Interestingly, the mRNAs encoding the STAT3-activating cytokine IL-6 and its alpha receptor (IL-6R) were significantly increased in the hearts of infiltrated mice (Fig 8A and Supporting Information Fig S2). Additionally, the expression of the IL-6R mRNA was significantly increased in the liver of both infiltrated and non-infiltrated mice (Fig 8A), where it may lead to amplified IL-6 signalling. Accordingly, IL-6 neutralization by means of anti-IL-6R antibodies could rescue 70% of the treated mice for up to 60 days of age (Fig 8B). Surviving mice showed virtually no auto-antibodies and significantly reduced C3 levels, heart infiltration, fibrosis and STAT3 phosphorylation, in keeping with the idea that inflammation, auto-immunity and C3 up-regulation are all dependent on IL-6 signalling and STAT3 activity (Fig 8C–F).

### IL-6 and C3 serum levels are highly correlated with heart STAT3 activation in human myocarditis

**Figure 9:**
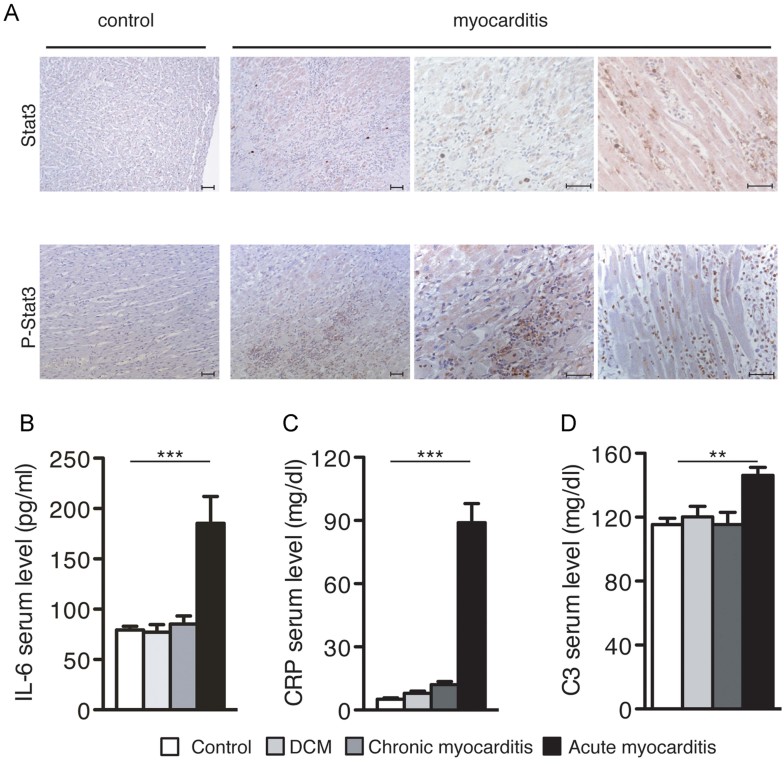
STAT3 phosphorylation and elevated circulating IL-6, CRP and C3 levels in human myocarditis. A. Immunohistochemical analysis. Hearts from human patients with myocarditis or from non-myocarditis controls, using antibodies detecting total (STAT3) or tyrosine-phosphorylated (P-STAT3) STAT3. Pictures are representative of three to five different patients. Scale bar: 50 µm. B. IL6 ELISA. Circulating IL-6 (mean ± SEM) levels were measured in the serum from healthy individuals (white bars, *n* = 10), or from patients with dilated cardiomyopathy (DCM, light grey bars, *n* = 9), chronic myocarditis (dark grey bars, *n* = 8) and acute myocarditis (black bars, *n* = 7). ***p < 0.0001 (Healthy *vs* Acute, DCM *vs* Acute, Chronic *vs* Acute). C. CRP ELISA on the same samples as in (B). ***p < 0.0001 (Healthy *vs* Acute, DCM *vs* Acute, Chronic *vs* Acute). D. C3 ELISA on the same samples as in (B). **p = 0.0054 (Healthy *vs* Acute); *p = 0.026 (DCM *vs* Acute); **p = 0.0087 (Chronic *vs* Acute).

Acute STAT3 activation is known to be beneficial in myocardial infarction, but its improper activation may be detrimental to heart function (Hilfiker-Kleiner et al, 2010). Accordingly, heart samples from human myocarditis patients clearly showed a massive infiltration of inflammatory cells, associated with strong STAT3 activation as assessed by IHC with phospho-specific STAT3 antibodies (Fig 9A). Patients with acute myocarditis displayed elevated circulating IL-6 and C Reactive Protein levels (Fig 9B and C), which correlated with significantly higher circulating C3, compared to healthy controls or to samples from DCM or chronic myocarditis (Fig 9D). These observations suggest a correlation between inflammation, IL-6, STAT3 activity and C3 levels also in human myocarditis, particularly during the acute/inductive phase.

## DISCUSSION

STAT3 is well known to play both beneficial and pathogenic roles depending on its activation mode, that is tightly controlled as in physiological settings as opposed to aberrantly continuously active under pathological conditions such as chronic inflammation and cancer (Camporeale & Poli, 2012; Yu et al, 2009). In the heart, STAT3 was shown to be protective in myocardial infarction as well as in viral-induced myocarditis (Hilfiker-Kleiner et al, 2004; Lindner et al, 2012; Oshima et al, 2005; Yajima et al, 2006, 2011). Conversely, Hilfiker-Kleiner et al have reported that mice displaying heart-specific enhanced STAT3 activity due to the expression of a mutated intracytoplasmic sequence of mouse gp130 (gp130^Y757/fl^) showed excessive inflammatory responses and lethality upon myocardial infarction (Hilfiker-Kleiner et al, 2010). Continuous STAT3 activation is pathogenic also in our model, stressing the importance of a tight control over STAT3 activity. Differently from the gp130^Y757/fl^ mutants, our Stat3^C/C^ mice retain intact gp130 signalling and, importantly, display whole body, low grade constitutive STAT3 activity (Barbieri et al, 2010). This triggers the development of an aggressive form of acute immune-mediated myocarditis requiring both enhanced IL-6 activity and C3 up-regulation, as shown by the IL-6 and complement neutralization experiments.

Importantly, we could demonstrate that STAT3 is also a major player in the pathogenesis and progression of EAM. Indeed, EAM development was almost completely abrogated by treatment with the SF-1-066 STAT3 inhibitor. Moreover, STAT3 inhibition starting at the peak of heart inflammation showed therapeutic effects, as evidenced by the reduced diastolic dysfunction and by the failure of SF-1-066-treated mice to significantly modify either the MPI or the fractional shortening. Thus, continuous STAT3 activation appears to be an important pathogenic factor in the susceptibility to myocarditis, favouring its progression to DCM and heart failure. These findings are relevant to the human disease, as we could show that heart samples from acute myocarditis patients contain high levels of phosphorylated STAT3. These correlate with high systemic inflammation, as evidenced by the elevated levels of IL-6 and CRP, and significantly enhanced circulating C3 levels.

Th17 CD4^+^ cells are believed to play an important role in the pathogenesis of EAM, although recent studies with IL-17A-deficient mice have shown that these cells are dispensable in the inflammatory phase (Baldeviano et al, 2010), suggesting at the same time an exacerbating role for Th1 and Th2 cells (Cihakova & Rose, 2008). In keeping with the known crucial function of IL-6 priming and STAT3 activity in Th17 cells differentiation (Harris et al, 2007; Haruta et al, 2011; Nishihara et al, 2007; Zhou et al, 2007), we observed an expanded population of IL-17-expressing cells in the spleen of the Stat3^C/C^ mice, correlating with increasingly abundant IL-17 in the hearts of Stat3^C/C^ mice. Additionally, naïve Stat3^C/C^ T cells displayed a significantly enhanced ability to differentiate along the Th17 lineage *in vitro*, even in the absence of IL-6. This is the first demonstration that aberrantly constitutive STAT3 activity is sufficient, both *in vivo* and *in vitro*, to skew the differentiation of CD4^+^ T lymphocytes towards the Th17 lineage. Despite these observations, Th17 cells do not appear to play a major role in the heart disease of the Stat3^C/C^ mice, since IL-17A neutralization could only protect 25% of the mice and only marginally affected heart infiltration, fibrosis and α-myosin antibody levels. Interestingly, analysis of endomyocardial biopsies failed to detect IL-17-expressing T cells in samples from patients with both acute myocarditis and DCM, suggesting a minor role for Th17 cells in humans as well (Noutsias et al, 2011). CD4 depletion was more effective in rescuing the disease in Stat3^C/C^ mice, albeit less than IL-6R neutralization or complement depletion, perhaps reflecting the role of other CD4^+^ T cells populations. Indeed, both Th1 and Th2 cells have been reported to be able to concur to myocarditis pathogenesis in EAM, although Th-1-produced IFN-γ appears to be protective (Afanasyeva et al, 2001, 2005; Cihakova & Rose, 2008; Daniels et al, 2008). Interestingly, neither IL-17 neutralization nor CD4 depletion could alter the up-regulation of C3 observed in the Stat3^C/C^ mice.

The expansion of the CD11b^+^/Gr1^+^ cell population, already observed in the blood and BM of non-infiltrated animals, and whose accumulation in the heart occurs before CD4^+^ cells detection, is likely to contribute to disease pathogenesis. This disruption in myeloid cells homeostasis may participate in the initial damage as well as contribute to the production of inflammatory cytokines. Myeloid cells have been proposed to play a role in EAM (Blyszczuk et al, 2009; Cihakova & Rose, 2008) and may also contribute to enhance C3 local production in the heart (Botto et al, 1992; Tsukamoto et al, 1993).

The experiments with BM chimeras have shown that the fully fledged disease can only develop when both haematopoietic and non-haematopoietic cells display constitutive STAT3 activity, and both lethality and C3 deposition appear to correlate with the expression of the Stat3^C/C^ allele in non-haematopoietic cells. Among these, our results suggest a central role for the hepatocytes. Indeed, the observation that IL-6R expression is up-regulated in the livers of Stat3^C/C^ mice prior to disease onset suggests local amplification of IL-6 signalling, in agreement with the previously observed up-regulation of several liver acute phase mRNAs (Barbieri et al, 2010). Indeed, STAT3 is well known to be part of a pro-inflammatory loop involving IL-6 and NF-kB, thought to play an important role in inflammation-related cancer (Karin & Greten, 2005; Yu et al, 2009) as well as in auto-immunity (Camporeale & Poli, 2012). IL-6 signalling amplification may in turn explain the enhanced liver expression of the acute phase protein C3, leading to its early heart localization observed in the Stat3^C/C^ mice before disease development. Together with the rescue obtained upon IL-6R neutralization and complement depletion, and the knowledge that C3 plays a pathogenic role in the inductive phase of EAM, downstream of IL-6 (Eriksson et al, 2003; Kaya et al, 2001) our results suggest that IL-6/STAT3-mediated enhanced C3 production in the liver is instrumental in disease initiation. This may be further amplified by local production of C3 by the infiltrating cells themselves, as both myeloid and CD4^+^ T cells are known to be able to express this protein (Botto et al, 1992; Kwan et al, 2012; Tsukamoto et al, 1993). As C3 can act as a chemo-attractant, its presence in the heart is likely to initiate a feed-forward loop, progressively attracting increasing numbers of inflammatory cells, and leading to heart muscle damage.

Death directly linked to acute myocarditis can be a serious clinical problem, particularly in developing countries (Feldman & McNamara, 2000). Moreover, heart inflammation is a relevant component of many other cardiac diseases, and myocarditis is considered one of the leading causes of idiopathic DCM and heart failure. However, what factors determine the switch between disease resolution and progression are at present incompletely understood, and the ability to identify individuals at greater risk of developing aggressive heart inflammation and to predict clinical development would improve patients care. In this light, our results suggest the idea that pre-existing inflammatory conditions not localized to the heart, increasing C3 production via an IL-6-STAT3 loop in the liver, might play a relevant pathogenic role in the onset of immune-mediated myocarditis. Moreover, the therapeutic effects of STAT3 neutralization in EAM suggest that this factor may also play a role in myocarditis progression to DCM. This mechanism may be relevant for human acute myocarditis patients as well, where we were able to demonstrate strong cardiac STAT3 activation correlating with high circulating IL-6 and with significantly elevated C3 levels. Further clinical correlation studies will be needed to establish whether circulating C3 levels might become a useful tool to help predicting susceptibility to aggressive myocarditis, and possibly its progression to DCM and heart failure.

## MATERIALS AND METHODS

### Animals and analysis

Generation of the Stat3^C/C^ mice was previously described (Barbieri et al, 2010). The mice used were in the BALB/c background (F8 backcross). Mice were maintained in the transgenic unit of the Molecular Biotechnology Center (University of Turin) under a 12-h light-dark cycle and provided food and water *ad libitum*. Procedures were in conformity with national and international laws and policies as approved by the Faculty Ethical Committee. Mice were inspected and weighed every 3 days, and sacrificed by cervical dislocation.

### Flow cytometry

To isolate infiltrating cells, hearts were disaggregated with collagenase type 2 and protease type XIV (Sigma–Aldrich, St. Louis MO, USA) as described. A FACScalibur flow cytometer (BD Bioscience, Franklin Lakes, NJ USA) and the cell quest software were used, with FITC anti-Gr1 and PE anti-CD11b antibodies (BD Bioscience). Intra-cellular cytokine staining was performed upon PMA/ionomycin and Brefeldin A treatment, followed by labelling with FITC anti-CD4 mAb (BD Bioscience), fixation in 2% paraformaldehyde, permeabilization with 0.1% saponin-1% FCS-PBS, and incubation with cytokine-specific mAbs (BD Bioscience).

### *Ex vivo* cytokines production

Splenocytes were incubated in RPMI medium (Invitrogen, Carlsbad CA, USA) supplemented with 10% FBS (Invitrogen), penicillin and streptomycin and stimulated as previously described (Veldhoen et al, 2006) followed by intracellular flow cytometry.

### *In vitro* T-cell differentiation assay

CD4^+^ naïve T cells were isolated from the spleens using the CD4^+^ CD62L^+^ T-cell isolation kit II (Miltenyi Biotec, Bergisch Gladbach, Germany). Cells were cultured in anti-CD3, anti-CD28 (BD Bioscience)-coated 24 multi-wells plates for 3 days in RPMI 1640 containing 10% heat-inactivated FCS, 50 µM 2-mercaptoethanol, 2 mM L-glutamine, 10 mM HEPES, 1 mM sodium pyruvate, 100 units/ml penicillin and 100 µg/ml streptomycin (Veldhoen et al, 2006) (Invitrogen) treated with 5 ng/ml TGF-β (Peprotech, Rocky Hill, NJ, USA), with 10 µg/ml anti-IFN-γ (BD Bioscience) and with or without 20 ng/ml IL-6 (Peprotech, Rocky Hill, NJ, USA) for Th17 differentiation, with 20 ng/ml IL-12, 20 ng/ml IL-2 and with 10 µg/ml anti-IL-4 (Peprotech, Rocky Hill, NJ, USA) for Th1 differentiation, with 50 ng/ml IL-4, 20 ng/ml IL-2 (Peprotech, Rocky Hill, NJ, USA) and 10 µg/ml anti-IFN-γ for Th2 differentiation, without cytokines for Th0 condition followed by IL-17 or IFN-γ or IL-4 intracellular staining and flow cytometry.

### Experimental auto-immune myocarditis

Eight-weeks old BALB/c mice were immunized essentially as described (Sonderegger et al, 2006) with the α-myosin heavy chain peptide Myhc-α_614–634_ (AnaSpec corporation, Fremont, CA, USA) or saline solution, emulsified 1:1 in Freund's complete adjuvant (CFA; Difco Laboratories, Detroit, Michigan, USA). 3 mg/kg of the STAT3 inhibitor SFI-066 (Zhang et al, 2010, provided by Prof. P. Gunning) were injected i.p. every 3 days starting from day 0 or day 21 of immunization. Appropriately diluted DMSO was used as control.

### ELISA assays

Anti-cardiac α-myosin IgG1 were measured upon coating with 3 µg/ml of cardiac myosin prepared as described (Shiverick et al, 1975), followed by incubation with mouse sera and HRP-labelled rat anti-mouse IgG1 (BD Bioscience). Murine C3 was captured with a polyclonal goat-IgG to mouse C3 and revealed with the same antibody peroxidase-conjugated (Cappel, ICN Pharmaceuticals, Costa Mesa, California, USA). SAP (Serum Amyloid P Component, Normal Mouse standard, Calbiochem, San Diego, CA, USA), was used as a standard according to manufacturer's instructions. Human CRP was determined by Beckman Synchron LX-20 analysis. Human C3 was measured by nephelometry with a goat anti-human antiserum.

### Lymphocytes proliferation assay

CD4^+^ T cells were isolated from spleens of immunized mice using the CD4 (L3T4) MicroBeads mouse kit (Miltenyi Biotec), and co-cultured for 72 h with 200,000 mitomycin A-treated syngeneic splenocytes (APCs) pre-cultured with Myhc-α_614–634_ peptides (1 mg/10^6^ cells). Cells were then pulsed with 0.5 Ci of [^3^H] methyl-thymidine (Perkin Elmer, MA, USA) for 16 h and proliferation evaluated by thymidine incorporation.

**The paper explained**

**PROBLEM**

Myocarditis, or inflammation of the heart muscle, is linked to the development of heart auto-immunity and can progress to dilated cardiomyopathy and heart failure in as much as one third of the cases. Disease management lacks of reliable diagnostic procedures and of specific therapeutic strategies, particularly because little is know of the factors determining the switch between disease resolution and progression.

**RESULTS**

Both IL-6 and IL-17 are thought to play a pathogenic role in auto-immune diseases and in particular in immune-mediated myocarditis. We show that pharmacological inhibition of the transcription factor STAT3, the main mediator of IL-6 functions and also essential for Th17 cells differentiation, abolishes the development of Experimental Auto-immune Myocarditis in mice, and can even act therapeutically when administered at disease peak, reducing the degree of cardiac dysfunction. This effect correlates with impaired myosin-specific CD4^+^ T lymphocytes responses and with reduced production of the complement component C3, a known pathogenic factor in the early phases of the disease. We then demonstrate that systemic constitutively active STAT3 is sufficient to trigger an aggressive form of immune-mediated myocarditis requiring STAT3-mediated up-regulation of C3 in the liver, and correlating with Th17 cells expansion. Indeed, disease development can be prevented by both depleting C3 and neutralizing IL-6R, whose signalling is amplified in the liver. In contrast, Th17 cells appear to only play a limited role. This mechanism may be relevant for the human disease, where we were able to demonstrate strong cardiac STAT3 activation correlating with high circulating IL-6 and with significantly elevated C3 levels in acute myocarditis patients.

**IMPACT**

IL-6 production and STAT3 activity are a hallmark of inflammation, acute or chronic, which impinges on the liver triggering the up-regulation of several plasma proteins including C3. Our data suggest that pre-existing inflammatory conditions involving IL-6/STAT3-mediated activation of the liver inflammatory response and C3 up-regulation may represent one of the factors determining the progression of myocarditis to DCM and heart failure. Further clinical correlation studies will be needed to establish whether circulating C3 levels might become a useful tool to help predicting susceptibility to aggressive myocarditis, and possibly its progression to DCM and heart failure.

### BM transplantation

Three weeks (Stat3^C/C^) or 8 weeks (Stat3^WT/WT^) old mice were subjected to lethal total body irradiation (2.5 or 5 Gy, respectively) administered twice with 3 h interval, followed by the IV injection of 10 × 10^6^ BM-derived cells from donor mice 1 h post-irradiation. Transplantation rate was assessed by quantitative Sybr Green PCR on peripheral blood genomic DNA. Chimeric animals with <80% of circulating donor cells were excluded from the analysis.

### *In vivo* depletion and IL-6 administration

Stat3^C/C^ mice were injected i.p. with 100 µg of anti-CD4 mAbs (Ibridoma GK1.5) or with 100 µg of purified rat mAb anti-mIL-17A, which recognizes both IL-17A and the IL-17A/F heterodimer (Rat IgG, R&D system, Minneapolis, MN, USA) or s.c. with 240 µg of the anti-IL-6Ralpha 15A7 mAb (Vink et al, 1990), once a week starting from 2 weeks of age. Control mice received isotype-matched IgG rat mAb (R&D system). CD4 depletion was assessed by flow cytometry on peripheral blood.

1 U/mouse of CVF (Sigma–Aldrich) was injected i.p. every 2 day for 10 days starting from day 21 after birth as previously described (Kaya et al, 2001).

### Histological and immunohistochemical analysis

Hearts were fixed in 4% paraformaldehyde for 24 h, paraffin-embedded and sections stained with haematoxylin–eosin or incubated overnight with rat anti-mouse CD18 (BMA Biomedicals, Augst, CH) followed by biotinylated or HRP anti-rat IgG (Dako Cytomation, Carpinteria, CA, USA). Antibodies for MAC3 and CD4 (BD Bioscience), MPO (Dako Cytomation), STAT3 (Santa Cruz Biotechnology, Santa Cruz CA, USA) and P-STAT3 (Cell Signaling Technology, Danvers MA, USA) were used on frozen sections after cold acetone fixation. Collagen fibres were evidenced by Picrosirius red staining (Fluka-Sigma–Aldrich). Quantification was performed using Metamorph software (Universal Imaging Corporation), averaging at least eight fields per sample.

### Immunofluorescence and confocal microscopy

Hearts were fixed in 4% paraformaldehyde for 4 h, dehydrated in 30% sucrose overnight and frozen in OCT. Sections (40 µm) were permeabilized with 0.3% Triton X-100 in PBS and incubated with primary antibodies for IL-17, CD4, Gr1 (BD Bioscience), C3 (Abcam, Cambridge, UK), IgG (Dako Cytomation), α-actinin and cx43 (Sigma–Aldrich), followed by Cy3 or AlexaFluor labelled secondary antibodies (Sigma–Aldrich). Rat adult cardiomyocytes were fixed in 4% paraformaldehyde and stained with mouse sera, followed by AlexaFluor-labelled secondary antibody (Sigma–Aldrich). Images were acquired using an Olympus Fluoview 200 laser scanning confocal system (Olympus, Inc., Germany) mounted on an inverted IX70 Olympus microscope, equipped with a 60X Uplan Fl (NA 1.25) oil-immersion objective. Image processing and analysis were performed with ImageJ software (Rasband WS, Image J, US National Institutes of health, Bethesda). The Cell Counter plugin was used to quantify cell numbers, while for intensity quantification of staining we used images acquired with the same brightness/contrast parameters.

### Real-time PCR

Total RNA was prepared with the PureLink Micro-to-Midi total RNA Purification System (Invitrogen). qRT-PCR reactions were performed as previously described using the Universal Probe Library system (Roche Diagnostic, Mannheim, Germany). Probes and oligonucleotide sequences used are reported in the Supporting Information Table S3. The 18S rRNA pre-developed TaqMan assay (Applied Biosystems, Carlsbad, California, USA) was used as an internal control.

### Western blot, immunoprecipitation and mass spectrometry

Total protein extracts (Maritano et al, 2004), were fractionated on SDS–PAGE and transferred to a polyvinylidene difluoride membrane (Millipore, Schwalbach, Germany). For immunoprecipitation, protein extracts were pre-cleared with protein G/A beads (GE Healthcare, New Jersey, USA), then precipitated with mouse serum, Myh-6 (a kind gift from Prof. Stefano Schiaffino, University of Padua) and My-BPC3 (Santa Cruz Biotechnology) antibodies and protein G beads, overnight at 4°C. MALDI mass spectrometry was performed as described.

### Echocardiography

Echocardiography was performed in conscious mice with a GE System 5 echocardiograph. Stat3^C/C^ mice were anaesthetized with 5 mg/kg of Zoletil 100 (Virbac, Milan, Italy), instead BALB/c mice with EAM with isofluorane. Mice were analysed with a Vevo2100 High Resolution Imaging System (Visual Sonics Inc, Toronto, Ontario, Canada) with the transducer MS550D. From the 2D long-axis were derived left ventricle M-mode: eft ventricle systolic and diastolic diameters (LVES and LVED), systolic and diastolic septum and posterior wall thickness (IVST and PWT) were measured. FS was calculated as (LVED-LVES)/LVED %. Pulsed Doppler on mitral flow was registered and E, A velocities and DT were measured. The ratio E/A were calculated. By tissue Doppler at the basal level of lateral wall were collected *E*′ and *A*′ velocities and their ratio *E*/*E*′. Diastolic function was defined as normal if *E*/*A* > 1, *E*′/*A*′ > 1, DT < 20 ms; I degree of dysfunction if *E*/*A* < 1, *E*′/*A*′ < 1, DT > 20 ms; II degree of dysfunction if *E*/*A* > 1, but *E*′/*A*′ < 1 and DT > 20; III degree of dysfunction if *E*/*A* > 2 and DT < 20 ms with reduced *E*′ and *A*′ velocities.

### Statistics

The unpaired two-tail Student's *t*-test with Welch's correction was used for comparisons between two groups of samples and in all echocardiographic analysis. One-way analysis of variance (ANOVA) with Tukey's HSD *post hoc* test was used for comparing three or more groups. A two-way analysis of variance (ANOVA) with Tukey's HSD *post hoc* test was used to analyse animal weights and *in vitro* Th17 differentiation. The Logrank Mantel-Cox test was used for significance of survival curves. *p*-Values lower or equal to 0.05 were considered significant.

## Author contributions

VP designed the project and the experiments, analysed data and wrote the paper; AC designed and performed experiments, analysed data and wrote the paper; FM performed Th1/Th2/Th17 and EAM experiments; RL and SH made conceptual contributions to the studies; SFo performed confocal microscopy and RL analysed confocal data; PC and MF performed IHC analyses; SH and AP analysed human samples; MM performed echocardiography studies; RC performed the histopathological analysis and OJ the mass spectrometry; SFl, BP and PG synthesized and provided the SF-1-066 inhibitor.

## Notes

As a service to our authors and readers, this journal provides supporting information supplied by the authors. Such materials are peer reviewed and may be re-organized for online delivery, but are not copy-edited or typeset. Technical support issues arising from supporting information (other than missing files) should be addressed to the authors.
