# Supplementary material for: STAT3 activity is necessary and sufficient for the development of immune-mediated myocarditis in mice and promotes progression to dilated cardiomyopathy
Source: EMBO Mol Med. 2013 Mar 5;5(4):572–90. doi: 10.1002/emmm.201201876 (PMC3628107; doi:10.1002/emmm.201201876)
Supplement: Supplementary file 1 [file emmm0005-0572-sd1.pdf]

STAT3 activity is necessary and sufficient for the development of immune-mediated myocarditis in mice and promotes progression to dilated cardiomyopathy

Annalisa Camporeale, Francesca Marino, Anna Papageorgiou, Paolo Carai, Sara Fornero, Steven Fletcher, Brent D.G. Page, Patrick Gunning, Marco Forni, Roberto Chiarle, Mara Morello, Ole Jensen, Renzo Levi, Stephane Heymans, Valeria Poli

Corresponding author: Valeria Poli, University of Turin -

Review timeline:

Submission date:	14 August 2012
Editorial Decision:	15 September 2012
Revision received:	22 December 2012
Editorial Decision:	20 January 2013
Accepted:	20 January 2013

Transaction Report:

(Note: With the exception of the correction of typographical or spelling errors that could be a source of ambiguity, letters and reports are not edited. The original formatting of letters and referee reports may not be reflected in this compilation.)

Editor: Anneke Funk / Roberto Buccione

1st Editorial Decision 15 September 2012

Thank you for the submission of your manuscript "Systemic constitutively active STAT3 triggers the onset of immune-mediated myocarditis: implications for the development of severe disease upon heart inflammation" to EMBO Molecular Medicine. We have now heard back from the three referees whom we asked to evaluate your manuscript. You will see that the referees find the topic of your manuscript potentially interesting. However, they also raise significant concerns on the study, which should be addressed in a major revision of the manuscript.

In particular, reviewer #3 highlights that it remains unclear whether the reduced life span of the Stat3C/C mice in deed results from myocarditis. Importantly, both Reviewer #2 and #3 note that the link between Th17 involvement, Stat3C/C and IL-6 should be strengthened.

Given the balance of these evaluations, we feel that we can consider a revision of your manuscript if you can convincingly address the issues that have been raised within the time constraints outlined below.

Revised manuscripts should be submitted within three months of a request for revision. They will otherwise be treated as new submissions, unless arranged otherwise with the editor.

I look forward to seeing a revised form of your manuscript as soon as possible.

***** Reviewer's comments *****

Referee #1 (Comments on Novelty/Model System):

Camporeale et al. present a great paper, which delineates in an integrative manner the mechanistic role of STAT3 activation in autoimmune myocarditis induction. Their findings also close a gap between our current understanding of the role of Th17 cell differentiation and our view on the role of IL-6 in Experimental Autoimmune Myocarditis. In addition, the study points to a novel therapeutic strategy for inflammatory heart diseases.
The paper is fluently written, and easy to follow. All major conclusions are supported by the presented data, and most of the figures appear in excellent quality (unfortunately I cannot find the Supporting Information Table II with the ECHO data). There are minor concerns, which should be addressed by the authors.

Referee #1 (General Remarks):

Camporeale et al. present a great paper, which delineates in an integrative manner the mechanistic role of STAT3 activation in autoimmune myocarditis induction. Their findings also close a gap between our current understanding of the role of Th17 cell differentiation and our view on the role of IL-6 in Experimental Autoimmune Myocarditis. In addition, the study points to a novel therapeutic strategy for inflammatory heart diseases.
The paper is fluently written, and easy to follow. All major conclusions are supported by the presented data, and most of the figures appear in excellent quality (unfortunately I cannot find the Supporting Information Table II with the ECHO data). There are minor concerns, which should be addressed by the authors (see below):

1.Dilated cardiomyopathy refers to an end stage heart failure phenotype, which CAN result from myocarditis. This phenotype includes dilated hearts with wall thinning, fibrosis, altered cardiomyocyte structure, impaired function but only minimal inflammation (=inflammatory Dilated Cardiomyopathy or iDCM). The Experimental Autoimmune Myocarditis model mirrors aspects of iDCM development, but this phenotype becomes obvious not earlier than 35 to 40 days after immunization and should not be mixed up with the inflammatory phenotype associated with extensive cardiac infiltrates at the peak of disease (around day 21 in the EAM model). Mechanisms promoting the transition of acute myocarditis into an end-stage heart failure phenotype are of major interest because they are the most promising targets for potential treatment strategies in humans. From this point of view the STAT3C/C mice represent a phenotype of ongoing myocarditis BUT CLEARLY NOT a phenotype of end stage heart failure. This must be clearly stated. From this point of view I would also omit the ECHO data (which I have not found): (i) they do not add to the paper, (ii) the used anesthesia protocol is not convincing (lower heart rate reflects deeper anesthesia, so you cannot exclude that the STAT3C/C are more susceptible to the cardiodepressive effects of your anesthetics), and (iii) the equipment as described in the method section is anyway not up-to-date for mouse echocardiography.
In the context of the above mentioned criticism I also miss a more thorough discussion and the citation of several recent key papers in the introduction, as well as in the discussion section (Barin JG, et al (2012) Eur J Immunol 42(3):726-736; Kania G, et al (2009) Circ Res 105:462-470; Blyszczuk P, et al (2009) Circ Res 105: 912-920; Afanasyeva M, et al. (2005) Proc Natl Acad Sci U S A 102: 180-185).

2.Figure 1B: the reviewer would appreciate to see a representative section covering the whole left ventricle at low magnification together with the hiher resolution image.

3.What is the effect of STAT3 inhibition AFTER day 21 (injections starting at day 21 until day 39, evaluation day 42? Does it prevent pathological remodeling and heart failure? In other words: is the suggested treatment a potential strategy for ACUTE myocarditis only?

4.Autoantibodies contribute to human myocarditis and there are antibody mediated experimental myocarditis model. Nevertheless, the EAM model is CD4 T cell mediated and works also (even better) in B cell deficient mice: from this point of view autoantibody titers are not appropriate surrogate markers for disease severity. They are however important, as they mirror epitope

spreading in the transgenic model.

5.Figure 5F and G: the text in the result section is misleading, as it suggests on page 7 that chimera were compared to "...non -transplanted STAT3C/C mice". Figure 5 and corresponding legends imply that controls were STAT3C/C in STAT3C/C chimera (i.e. the appropriate control!). Please make clear!

Referee #2:

The development of autoimmune myocarditis and subsequent dilated cardiomyopathy has been associated with IL-6/STAT3 signaling, C3 complement generation, anti-cardiac auto-antibody production, and effector functions of the Th17 lymphocytic lineage. Here, Camporeale et al. examine the effects of negative and positive STAT3 modulation on the development of autoimmune myocarditis. Inhibition of STAT3 with the STAT3 inhibitor, SF-1-066, protects BALB/c mice from myosin peptide-induced autoimmune myocarditis, as demonstrated by significant reduction in immune cell infiltration and cardiac fibrosis; serum C3 complement and anti-myosin IgG antibody levels were abrogated, and in vitro myosin-specific CD4+ T lymphocyte proliferation was blunted. On the contrary, knock-in mice that expressed constitutively active STAT3 (STAT3C) were found to spontaneously develop aggressive auto-immune myocarditis. Interestingly, the authors showed that despite diffuse systemic expression of STAT3C, STAT3C/C mice exhibited histopathological changes predominantly in the heart. The authors characterized the immunological aberrations in the STAT3C/C mice in the context of autoimmune myocarditis. The authors demonstrated that spontaneous autoimmune myocarditis in the STAT3C/C mice was associated with STAT3C-driven up-regulation of hepatic C3 complement, specific anti-cardiac antigen auto-antibody generation, IL-6 up-regulation, and expansion of the Th17 lymphocytic lineage. In the STAT3C/C mice, blockade of the IL-6 pathway with anti-IL-6Ra 15A7 mAb, depletion of CD4+ lineage with anti-CD4 Ibridoma GK1.5 mAb, or C3 complement depletion with cobra venom factor rescued STAT3C/C mice to varying degrees from spontaneous autoimmune myocarditis. The authors also showed through reciprocal bone marrow grafting that STAT3C activity in both hematopoietic and non-hematopoietic compartments contributed to the spontaneous development of autoimmune myocarditis. Finally, the authors report that patients with acute myocarditis had STAT3 activation and C3 up-regulation. They propose that IL-6/STAT3 activity and C3 complement levels might be considered as prognostic biomarkers for the progression of myocarditis to severe heart failure.
Several Questions Arise:
1) Given that STAT3 is downstream of several signaling molecules, including IL-6, and the critical role of STAT3 and IL-6 in Th17 differentiation, the authors showed in Figure 5A that spleens from STAT3C/C knock-in mice have higher frequency of IL-17 producing cells (Th17). For completeness, have the authors examined the effects of STAT3C knock-in on the expression of other Th17-skewing biomarkers such as IL-23, TGF- , and ROR- T?
2) Presumably, constitutively active STAT3 (STAT3C) would override the IL-6 requirement. In Figure 5B, the authors show that in vitro differentiation of STAT3C/C cells preferentially skewed toward IL-17-producing CD4+ Th17 cells. Differentiation was done in the presence of TGF- , IL-6, and anti-IFN- antibody. Do STAT3C/C cells still need IL-6 for Th17 differentiation? It would strengthen the paper if the authors could show that STAT3C overrides the IL-6 requirement for Th17 differentiation, i.e., differentiate cells in the absence of IL-6.
3) Figure 5G shows data from bone marrow chimera study. It would be important to have a C C irradiated/bone marrow transfer group for direct comparison.
4) It is premature to suggest that the presence of IL-6 and C3 complement in acute myocarditis would potentially serve as prognostic biomarkers for subsequent heart failure. This claim is still purely speculative. Also, the abstract and introduction were written to imply the identification of biomarkers prognostic of the transition from acute myocarditis to dilated cardiomyopathy/heart failure. Please modify accordingly.
5) The authors examined various anatomical tissues in STAT3C/C knock-in mice (Supplemental Table I). Interestingly, STAT3C/C knock-in mice exhibited histopathology predominantly in the heart. The authors could discuss why the heart is predominantly affected by the STAT3C knock-in. Is STAT3 normally highly expressed in the heart in wild-type mice?
6) Previously, it has been shown by others that cardiomyocyte-specific deletion of STAT3 was detrimental in various cardiotoxicity models, suggesting that STAT3 was cardioprotective in the

cardiac myocytes, perhaps through a pro-survival mechanism. The authors showed that systemic expression of STAT3C, on the other hand, caused spontaneous development of myocarditis. Taken together, these findings suggest that fine-tuned molecular regulation of STAT3 activity is critical to health. How might STAT3C be promoting inflammation?
7) Is STAT3C expression affecting the t1/2 of immune cells?
8) Can the STAT3 inhibitor, SF-1-066, inhibit STAT3C activity? Can STAT3 inhibition in vivo in STAT3C/C mice protect them from spontaneous autoimmune myocarditis? If so, this would provide further support for the specificity of STAT3C knock in.
Minor comments:
1) The manuscript requires appropriate grammatical corrections.
2) It is usually helpful to the reader if the results were described systematically in the order that they appear in the Figures.
3) In the methods section, under Animals and analysis, please indicate the reference(s) where the generation of STAT3C/C mice was described. Please indicate the genetic background of the STAT3C/C mice.
4) In the methods section, under Experimental auto-immune myocarditis, please indicate the reference(s) where the immunization protocol was described. Also, please indicate the Route of delivery of the STAT3 inhibitor, SF-1-066. Please also indicate where the STAT3 inhibitor SF-1-066 was obtained from.
5)In the supporting information Figure 2, levels of pro-inflammatory cytokines/chemokines are shown for the various mice. Please indicate which IL-17 subtype was specifically measured. Please also include data for IFN- , IL-10, and TGF- in the figure. Similarly, in Supporting Information Table III, please indicate which IL-17 subtype the primer sequences are for.

Referee #3:

The manuscript by Camporeale and colleagues provide intriguing evidence that homozygous mice ubiquitously expressing a constitutive version of Stat3 (Stat3C/C) spontaneously develop immune-mediated myocarditis. Depletion studies furthermore suggest a causal involvement of CD4 lymphocytes, interleukin 6 and complement C3. In addition, the authors also make correlative observations between the development of myocarditis, skewed Th17 polarization and excessive production of (auto-)antibodies against Myh6 and MyBP-C3 as well as infiltration of the myocardium with CD11+ myelocytes.

This is a clearly written manuscript with, mostly, easy to understand figures. The authors put their findings in context with clinical observations in human suffering from acute myocarditis as well as with highly relevant, earlier observation by Hilfiker-Kleiner in mice, where the latter group observed increased inflammation in response to myocardial infarction in mice with excessive activation of endogenous Stat3. Thus, the observations presented here are not entirely unexpected, although in the present work the phenotype occurs spontaneously in Stat3C/C mice rather than in response to an insult in gp130 mice in the former study. It therefore would seem appropriate if authors could discuss this difference in particular with a focus of a possible threshold effect for Stat3 activation. This is a pertinent issue in light of the authors' intriguing observation that intrinsic Stat3C activity is insufficient to confer disease, but requires additional IL6-dependent (and hence also Stat3 dependent ?) amplification. Does administration of IL6 to Stat3C/wt mice also induce disease? Do anti-IL6 treated Stat3C/C mice have less P-Stat3?

Other issues that require clarification:
1) For many treatments discussed here survival is taken as endpoint. However, the bone-marrow chimera studies clearly suggest that the extent of myocarditis and survival does not correlate given that C>wt and wt>C mice both develop mild disease, but wt>C show the same reduced lifespan as naive Stat3C/C mice. How do authors know that the severely reduced life span results from myocarditis rather than systemic C3-dependent inflammation? Do C3 levels correlate with survival amongst different groups of bone marrow chimeras? This reviewer notes similar H&E, CD18 and picrosirius stains for antiCD4, wt>wt and wt>C mice although these groups show different survivals - why? What is the age of the mice from which histology is show in Figs 5E/G ?
2) The Abstract/Discussion/Paper Explained sections give undue emphasis on a Th17 involvement when authors only show correlation with enhanced serum IL17 and enhanced in vitro Th17

polarization. Does IL17 depletion replicate the authors' observations with anti-IL6 antibodies?
3) Details referred to in the text can not readily be detected on some photomicrographs (ie. in Fig 8 "lymphocytes" and the cell-types staining positive for P-Stat3). Fig 5G should have an additional row documenting the phenotype of Stat3C/C mice, or even better C>C mice, for comparison - this magnification also makes it not possible to discern the cell types that stain for CD18. Please show high magnification.
4) Why do the Westernblots (Fig 4D) show simultaneously bands for Myh6 and MyBP-C3 when only blotted for one of these proteins in the 3rd and 4th panel?
5) The representation of the Kaplan-Meier plots is confusing. Conventionally the position of a data point on the x-axis indicates the death of an animal thereby reducing the proportion of surviving animals to the next lower level as indicated by a vertical line. Accordingly the last data point is always above the axis. In Fig 7B the curves for the anti-IL6 treated animals confusingly remains at 100% after the first data point and this curve shows only 4 data points although n=7 in corresponding legend. From the data shown, it seems not valid to claim a significant difference between the IgG and anti-IL6 treated groups when the only difference is due to a single long surviving mouse in the anti-IL6 group.
6) The quantification of data in Figs 3A-C does not show a consecutive occurrence of CD4 accumulation (causally involved in mediating disease in Stat3C mice) and cx43 stain. Therefore authors should not make a causal connection between "inflammation" and "connexin 43 disorganization" in the Results section. Similarly, authors refer in the Result sections to elevated IL6 level in humans with acute myocarditis; they should provide data for that for the samples the analyzed in Fig 8.

Minor comments
1) All photomicrographs should have scale bars; referring to magnification is meaningless, because the size of the reproduced photomicrographs is different from the ones collected on the microscope.
2) Authors should state what Picrosirius red stains.
3) In the first paragraph of the Discussion, authors refer to an incorrect phenotype (gp130Y757/Y757) for the mice used in the Hilfiker-Kleiner study; these mice were gp130Y757/flox.
4) In Supporting Fig S2 N-values should be given, and the use of black and grey in the histograms in inconsistent with that in rest of manuscript (i.e. Fig 2, 6, 7)

1st Revision - authors' response 22 December 2012

Referee #1

Camporeale et al. present a great paper, which delineates in an integrative manner the mechanistic role of STAT3 activation in autoimmune myocarditis induction. Their findings also close a gap between our current understanding of the role of Th17 cell differentiation and our view on the role of IL-6 in Experimental Autoimmune Myocarditis. In addition, the study points to a novel therapeutic strategy for inflammatory heart diseases. The paper is fluently written, and easy to follow. All major conclusions are supported by the presented data, and most of the figures appear in excellent quality (unfortunately I cannot find the Supporting Information Table II with the ECHO data). There are minor concerns, which should be addressed by the authors.

1. Dilated cardiomyopathy refers to an end stage heart failure phenotype, which CAN result from myocarditis. This phenotype includes dilated hearts with wall thinning, fibrosis, altered cardiomyocyte structure, impaired function but only minimal inflammation (inflammatory Dilated Cardiomyopathy or iDCM). The Experimental Autoimmune Myocarditis model mirrors aspects of iDCM development, but this phenotype becomes obvious not earlier than 35 to 40 days after immunization and should not be mixed up with the inflammatory phenotype associated with extensive cardiac infiltrates at the peak of disease (around day 21 in the EAM model). Mechanisms promoting the transition of acute myocarditis into an end-stage heart failure phenotype are of major interest because they are the most promising targets for potential treatment strategies in humans. From this point of view the $STAT3^{C/C}$ mice represent a phenotype of ongoing myocarditis BUT CLEARLY NOT a phenotype of end stage heart failure. This must be clearly stated. From this point

of view I would also omit the ECHO data (which I have not found): (i) they do not add to the paper, (ii) the used anesthesia protocol is not convincing (lower heart rate reflects deeper anesthesia, so you cannot exclude that the STAT3$^{C/C}$ are more susceptible to the cardiodepressive effects of your anesthetics), and (iii) the equipment as described in the method section is anyway not up-to-date for mouse echocardiography. In the context of the above mentioned criticism I also miss a more thorough discussion and the citation of several recent key papers in the introduction, as well as in the discussion section (Barin JG, et al (2012) Eur J Immunol 42:726-736; Kania G, et al (2009) Circ Res 105:462-470; Blyszczuk P, et al (2009) Circ Res 105: 912-920; Afanasyeva M, et al. (2005) Proc Natl Acad Sci U S A 102: 180-185).

A/ As suggested, we have stated that "*the described heart damage in the Stat3$^{C/C}$ mice clearly represents a phenotype of ongoing myocarditis*" (page 7, at the end of phenotype description). We have also removed the ECHO data referring to the heart rate, which we agree may well reflect deeper anesthesia in the Stat3$^{C/C}$ mice. However, since the fractional shortening is significantly reduced in the Stat3$^{C/C}$ mice, we still show this piece of data in Supplementary Table II (part of the Supporting Information file, which we hope will be visible to the referee this time). We thus state that the ongoing myocarditis is "*starting to lead to altered heart functionality as judged by echocardiography, since fractional shortening appeared to be significantly reduced (Supporting Information Table II)*" (Results section, page 7). Moreover, we have completed the description of the echocardiographic equipment in the Methods section, which we realized was indeed misleading. As suggested, we have cited Blyszczuk et al., 2009 and Afanasyeva et al., 2005, in the Discussion section.

2. Figure 1B: the reviewer would appreciate to see a representative section covering the whole left ventricle at low magnification together with the higher resolution image.

A/ We have included 2x magnification photomicrographs as requested (new Fig. 1B).

3. What is the effect of STAT3 inhibition AFTER day 21 (injections starting at day 21 until day 39, evaluation day 42? Does it prevent pathological remodeling and heart failure? In other words: is the suggested treatment a potential strategy for ACUTE myocarditis only?

A/ We thank the referee for what was indeed a very good suggestion. We have performed the experiment and the results are reported in the new Fig. 1, E-G and Supporting Information Fig. 1, C-E. The results clearly show that STAT3 inhibition starting at day 21 significantly reduces heart failure (see Results section, end of page 5 and beginning of page 6). These data integrate those obtained with the Stat3$^{C/C}$ mice, and lead us to modify accordingly the Abstract (…*pharmacological inhibition of STAT3 … can act therapeutically when administered at disease peak*); Discussion (page 11: *"Moreover, STAT3 inhibition starting at the peak of heart inflammation showed therapeutic effects, as evidenced by the reduced diastolic dysfunction and by the failure to significantly modify either the myocardial performance index or the fractional shortening. Thus, continuous STAT3 activation appears to be an important pathogenic factor in the susceptibility to myocarditis, favoring its progression to DCM and heart failure"*); and Paper explained sections (Results and Impact).

4. Autoantibodies contribute to human myocarditis and there are antibody mediated experimental myocarditis model. Nevertheless, the EAM model is CD4 T cell mediated and works also (even better) in B cell deficient mice: from this point of view autoantibody titers are not appropriate surrogate markers for disease severity. They are however important, as they mirror epitope spreading in the transgenic model.

A/ It is indeed true that the EAM model is not antibody-mediated. However, anti-myosin antibodies are a useful marker to follow disease development, as we have observed that their levels correlate

with the extent of inflammatory cells infiltration, both in the EAM and the transgenic model. Disease severity was always evaluated at sacrifice, by cytofluorimetric analysis of $CD11b^+$ infiltrating cells and by histological, histochemical and immunohistochemical analysis of infiltration and fibrosis.

5. Figure 5F and G: the text in the result section is misleading, as it suggests on page 7 that chimera were compared to "...non -transplanted $STAT3^{C/C}$ mice". Figure 5 and corresponding legends imply that controls were $STAT3^{C/C}$ in $STAT3^{C/C}$ chimera (i.e. the appropriate control!). Please make clear!

A/ We have included $STAT3^{C/C}$ in $STAT3^{C/C}$ chimeras as a control group in the new Fig. 6C-E and Supporting Fig. 3D-F, and clearly indicated it in the text and figures. Moreover, we have included new data showing that C3 deposition only occurs in $STAT3^{C/C}$ recipient mice, regardless of the genotype of the transplanted cells and correlating with premature death. This piece of data clearly shows that C3 production is contributed by non hematopoietic $STAT3^{C/C}$ cells, and that its accumulation in the heart may represent a crucial pathogenic factor in our model (Results section, page 9; Discussion section, page 12).

Referee #2

The development of autoimmune myocarditis and subsequent dilated cardiomyopathy has been associated with IL-6/STAT3 signaling, C3 complement generation, anti-cardiac auto-antibody production, and effector functions of the Th17 lymphocytic lineage. Here, Camporeale et al. examine the effects of negative and positive STAT3 modulation on the development of autoimmune myocarditis. Inhibition of STAT3 with the STAT3 inhibitor, SF-1-066, protects BALB/c mice from myosin peptide-induced autoimmune myocarditis, as demonstrated by significant reduction in immune cell infiltration and cardiac fibrosis; serum C3 complement and anti-myosin IgG antibody levels were abrogated, and in vitro myosin-specific CD4+ T lymphocyte proliferation was blunted. On the contrary, knock-in mice that expressed constitutively active STAT3 (STAT3C) were found to spontaneously develop aggressive auto-immune myocarditis. Interestingly, the authors showed that despite diffuse systemic expression of STAT3C, STAT3C/C mice exhibited histopathological changes predominantly in the heart. The authors characterized the immunological aberrations in the STAT3C/C mice in the context of autoimmune myocarditis. The authors demonstrated that spontaneous autoimmune myocarditis in the STAT3C/C mice was associated with STAT3C-driven up-regulation of hepatic C3 complement, specific anti-cardiac antigen auto-antibody generation, IL-6 up-regulation, and expansion of the Th17 lymphocytic lineage. In the STAT3C/C mice, blockade of the IL-6 pathway with anti-IL-6Ra 15A7 mAb, depletion of CD4+ lineage with anti-CD4 Ibridoma GK1.5 mAb, or C3 complement depletion with cobra venom factor rescued STAT3C/C mice to varying degrees from spontaneous autoimmune myocarditis. The authors also showed through reciprocal bone marrow grafting that STAT3C activity in both hematopoietic and non-hematopoietic compartments contributed to the spontaneous development of autoimmune myocarditis. Finally, the authors report that patients with acute myocarditis had STAT3 activation and C3 up-regulation. They propose that IL-6/STAT3 activity and C3 complement levels might be considered as prognostic biomarkers for the progression of myocarditis to severe heart failure.

Several Questions Arise:

1. Given that STAT3 is downstream of several signaling molecules, including IL-6, and the critical role of STAT3 and IL-6 in Th17 differentiation, the authors showed in Figure 5A that spleens from $STAT3^{C/C}$ knock-in mice have higher frequency of IL-17 producing cells (Th17). For completeness, have the authors examined the effects of STAT3C knock-in on the expression of other Th17-skewing biomarkers such as IL-23, TGF-b, and ROR-gT?

A/ We included the analysis of the requested biomarkers in new Fig. 5D, showing higher expression of both IL-23 and ROR-gT in the STAT3$^{C/C}$ mice.

2) Presumably, constitutively active STAT3 (STAT3C) would override the IL-6 requirement. In Figure 5B, the authors show that in vitro differentiation of STAT3C/C cells preferentially skewed toward IL-17-producing CD4^{+} Th17 cells. Differentiation was done in the presence of TGF-b, IL-6, and anti-IFNg antibody. Do STAT3$^{C/C}$ cells still need IL-6 for Th17 differentiation? It would strengthen the paper if the authors could show that STAT3C overrides the IL-6 requirement for Th17 differentiation, i.e., differentiate cells in the absence of IL-6.

A/ We performed the suggested experiment (new Fig. 5C), demonstrating that STAT3C expression in STAT3$^{C/C}$ naïve T cells can indeed override the IL-6 requirement for Th17 differentiation. The differentiation rate is however much lower than that observed in the presence of IL-6. This is not surprising, since it is known that the STAT3C mutant form still requires signal-induced phosphorylation to become fully active (Liddle FJ et al., Biochemistry 2006, 45:5599-605). We have observed that the levels of STAT3C phosphorylation detected in the tissues and cells of STAT3$^{C/C}$ mice are low as compared to those observed upon acute stimulation, while its signal-induced activation is prolonged (Barbieri et al., Cancer Res. 2010, 7:2558-67). It is therefore expected that IL-6 stimulation can still enhance STAT3C activity. We have included the statement that STAT3 activity is continuous, but low, in the Introduction section.

3) Figure 5G shows data from bone marrow chimera study. It would be important to have a C/C irradiated/bone marrow transfer group for direct comparison.

A/ We have included STAT3$^{C/C}$ in STAT3$^{C/C}$ chimeras as a control group in the new Fig. 6C-E and Supporting Fig. 3D-F, and clearly indicated it in the text and figures. Moreover, we have also included new data showing that C3 deposition only occurs in STAT3$^{C/C}$ recipient mice, regardless of the genotype of the transplanted cells and correlating with premature death. This piece of data clearly shows that C3 production is contributed by non hematopoietic STAT3$^{C/C}$ cells, and that its accumulation in the heart may represent a crucial pathogenic factor in our model (Results section, page 9; Discussion section, page 12).

4) It is premature to suggest that the presence of IL-6 and C3 complement in acute myocarditis would potentially serve as prognostic biomarkers for subsequent heart failure. This claim is still purely speculative. Also, the abstract and introduction were written to imply the identification of biomarkers prognostic of the transition from acute myocarditis to dilated cardiomyopathy/heart failure. Please modify accordingly.

A/ We have included new data (new Fig. 1 E-G and Supporting Information Fig. 1, C-E) showing that STAT3 inhibition starting at day 21 of EAM (the peak of heart inflammation) can act therapeutically, significantly reducing heart failure (Results section, pages 5 and 6). These data integrate those obtained with the Stat3$^{C/C}$ mice, suggesting that STAT3 activity is not only required for the onset of myocarditis in EAM, but that it is also involved in its progression to DCM. Accordingly, we have modified the title ("*STAT3 activity is necessary and sufficient for the development of immune-mediated myocarditis in mice and promotes progression to dilated cardiomyopathy*"), Abstract (…*pharmacological inhibition of STAT3 … can act therapeutically when administered at disease peak*); Discussion (page 11: *"Moreover, STAT3 inhibition starting at the peak of heart inflammation showed therapeutic effects, as evidenced by the reduced diastolic dysfunction and by the failure to significantly modify either the myocardial performance index or the fractional shortening. Thus, continuous STAT3 activation appears to be an important pathogenic factor in the susceptibility to myocarditis, favoring its progression to DCM and heart failure"*); and Paper explained sections (Results and Impact). Additionally, also the correlation between C3 heart deposition, myocarditis degree and premature death reported above for the bone-marrow chimeras (point 3, new Fig. 6 C-E and Supporting Fig. 3D-F) strengthens in our opinion the correlation between C3 and the aggressive myocarditis development. Nevertheless, we agree that the conclusion that C3 levels at the onset of acute myocarditis could serve as predictive markers for subsequent

heart failure is speculative, and re-phrased the text accordingly (see the last sentences in both the Abstract and Discussion sections).

5) The authors examined various anatomical tissues in STAT3$^{C/C}$ knock-in mice (Supplemental Table I). Interestingly, STAT3$^{C/C}$ knock-in mice exhibited histopathology predominantly in the heart. The authors could discuss why the heart is predominantly affected by the STAT3C knock-in. Is STAT3 normally highly expressed in the heart in wild-type mice?

A/ The predominant heart histopathology observed in the Stat3$^{C/C}$ mice is still incompletely understood. STAT3 levels are not particularly high in the heart of Stat3$^{C/C}$ mice (Barbieri et al., Cancer Res 2010, 70:2558-67). However, our data suggest that the main factor initiating heart inflammation is likely to be the STAT3-dependent amplification of IL-6 signalling in the liver and perhaps in myeloid cells, leading to up-regulated C3 production and to increased C3 circulating levels and heart deposition. Indeed, cardiomyocytes are known to be particularly sensitive to complement-mediated toxicity (Afanasyeva M, Rose NR. Am J Pathol. 2002, 161:351-7), and even a small initial damage can act as an inflammatory trigger. Additionally, the expanded myeloid cells population is also likely to contribute to the initiation and amplification of heart inflammation, since inflammatory cytokines can lead to cardiac dysfunction, inducing tissue damage and mediating fibrosis and cardiomyocyte hypertrophy (Marchant DJ Circ Res 2012, 110:126-44). We tried to state this more clearly in the Discussion section (pages 12-13).

6) Previously, it has been shown by others that cardiomyocyte-specific deletion of STAT3 was detrimental in various cardiotoxicity models, suggesting that STAT3 was cardioprotective in the cardiac myocytes, perhaps through a pro-survival mechanism. The authors showed that systemic expression of STAT3C, on the other hand, caused spontaneous development of myocarditis. Taken together, these findings suggest that fine-tuned molecular regulation of STAT3 activity is critical to health. How might STAT3C be promoting inflammation?

A/ It is well recognized that STAT3 is part of a pro-inflammatory positive loop involving IL-6 and NF-kB (Karin M. and FR Greten, Nat Rev Immunol 2005, 5:749-59; Yu H., Nat Rev Cancer 2009, 9:798-809), and indeed we have observed up-regulated expression of the IL-6R (Fig. 8A) and of acute phase protein mRNAs (Barbieri et al., Cancer Res. 2010, 70:2558-67) in the liver of the Stat3$^{C/C}$ mice. We point this out in the Discussion section, page 12. Additionally, STAT3 is required to mediate IL-6 functions and Th17 cells differentiation, and its aberrantly constitutive activity can thus promote chronic inflammation (Camporeale et al., 2012, 17:2306-26).

7) Is STAT3C expression affecting the t1/2 of immune cells?

A/ We performed new experiments showing that Stat3$^{C/C}$ CD4^{+} naïve T cells retain normal differentiation potential into both the Th1 and Th2 lineages (new Supporting Fig. 3 F-H), in contrast to the skewed Th17 differentiation observed both in vivo and in vitro. Interestingly, we have in contrast observed an expansion of Th2, but not Th1, cells in the spleen of Stat3$^{C/C}$ mice, suggesting that STAT3 constitutive activity may indirectly stimulate, possibly via IL-17 producing cells (Wakashin et al., Am J Respir Crit Care Med. 2008, 178:1023-32), differentiation towards the Th2 lineage. In turn, increased numbers of Th2 cells could contribute to myocarditis pathogenesis, as proposed by Afanasyeva et al., (Am J Pathol. 2001, 159:193-203).

8) Can the STAT3 inhibitor, SF-1-066, inhibit STAT3C activity? Can STAT3 inhibition in vivo in STAT3C/C mice protect them from spontaneous autoimmune myocarditis? If so, this would provide further support for the specificity of STAT3C knock in.

A/ Both the S3I-201 (Siddiquee et al., PNAS, 2007, 104:7391-6), and the BP1-102 (Zhang et al., PNAS, 2012, 19:9623-9628) STAT3 inhibitors, which are respectively the precursor (S3I-201) and

the derivative (BP1-102) of the SF-1-066 compound, have been shown to be unable to inhibit the STAT3C form. In our hands the SF-1-066 inhibitor was also not effective inhibiting STAT3 activity in $Stat3^{C/C}$ mice/cells, making the suggested experiment impossible.

Minor comments

1) The manuscript requires appropriate grammatical corrections.

A/ We have made our best to correct grammatical errors.

2) It is usually helpful to the reader if the results were described systematically in the order that they appear in the Figures.

A/ We have rearranged the order of the figures/text accordingly.

3) In the methods section, under Animals and analysis, please indicate the reference(s) where the generation of $STAT3^{C/C}$ mice was described. Please indicate the genetic background of the $STAT3^{C/C}$ mice.

A/ We have provided the requested information.

4) In the methods section, under Experimental auto-immune myocarditis, please indicate the reference(s) where the immunization protocol was described. Also, please indicate the Route of delivery of the STAT3 inhibitor, SF-1-066. Please also indicate where the STAT3 inhibitor SF-1-066 was obtained from.

A/ We have provided the requested information.

5) In the supporting information Figure 2, levels of pro-inflammatory cytokines/chemokines are shown for the various mice. Please indicate which IL-17 subtype was specifically measured. Please also include data for IFN-g, IL-10, and TGF-b; in the figure. Similarly, in Supporting Information Table III, please indicate which IL-17 subtype the primer sequences are for.

A/ The IL-17 subtype detected is IL-17A, and we have included the information where requested. We also have included data for IFN-g, IL-10, and TGF-b in the new Supporting Fig. 2.

<u>Referee #3</u>

The manuscript by Camporeale and colleagues provide intriguing evidence that homozygous mice ubiquitously expressing a constitutive version of Stat3 (Stat3C/C) spontaneously develop immune-mediated myocarditis. Depletion studies furthermore suggest a causal involvement of CD4 lymphocytes, interleukin 6 and complement C3. In addition, the authors also make correlative observations between the development of myocarditis, skewed Th17 polarization and excessive production of (auto-)antibodies against Myh6 and MyBP-C3 as well as infiltration of the myocardium with CD11+ myelocytes.

This is a clearly written manuscript with, mostly, easy to understand figures. The authors put their findings in context with clinical observations in human suffering from acute myocarditis as well as with highly relevant, earlier observation by Hilfiker-Kleiner in mice, where the latter group observed increased inflammation in response to myocardial infarction in mice with excessive activation of endogenous Stat3. Thus, the observations presented here are not entirely unexpected, although in the present work the phenotype occurs spontaneously in Stat3C/C mice rather than in response to an insult in gp130 mice in the former study. It therefore would seem appropriate if authors could discuss this difference in particular with a focus of a possible threshold effect for Stat3 activation. This is a pertinent issue in light of the authors' intriguing observation that intrinsic Stat3C activity is insufficient to confer disease, but requires additional IL6-dependent (and hence also Stat3 dependent ?) amplification. Does administration of IL6 to Stat3C/wt mice also induce disease? Do anti-IL6 treated Stat3C/C mice have less P-Stat3?

A/ The main difference between the model described by Hilfiker-Kleiner and colleagues and ours is that STAT3 constitutive activity occurs in every tissue as opposed to the combination of the gp130^{Y757} allele expression with the deletion of the wild type gp130 allele in gp130$^{Y757/fl}$ mice, which occurs specifically in the heart (Hilfiker Kleiner et al., Circulation 2010, 122,145-155). This is highlighted in the first paragraph of the Discussion section. As we point out in both the Results and Discussion sections, our data suggest that the main factor initiating heart inflammation is likely to be the STAT3-dependent amplification of IL-6 signalling in the liver and perhaps in myeloid cells, leading to up-regulated C3 production and to increased C3 circulating levels and heart deposition. On the other hand, we agree that there must be a threshold for STAT3 activation. Indeed, in new Fig. 5C we demonstrate that STAT3C expression in STAT3$^{C/C}$ naïve T cells can override the IL-6 requirement for Th17 differentiation. The differentiation rate is however much lower than that observed in the presence of IL-6. This is not surprising, since it is known that the STAT3C mutant form still requires stimulation to become fully active (Liddle FJ et al., Biochemistry 2006, 45:5599-605), and that the levels of STAT3C phosphorylation detected in the tissues of STAT3$^{C/C}$ mice are low as compared to those observed upon acute stimulation, while its signal-induced activation is prolonged (Barbieri et al., Cancer Res. 2010, 70:2558-67). We have included the statement that STAT3 activity is continuous, but low, in the Introduction section. In this light, it is therefore not surprising that intrinsic Stat3C activity is insufficient to confer disease, but requires additional IL6-dependent (and hence also STAT3 dependent) amplification. Indeed, it is well recognized that STAT3 is part of a pro-inflammatory positive loop involving IL-6 and NF-kB (Karin M. and FR Greten, Nat Rev Immunol 2005, 5:749-59; Yu H., Nat Rev Cancer 2009, 9:798-809), and indeed we have observed up-regulated expression of the IL-6R (Fig. 8A) and of acute phase protein mRNAs (Barbieri et al., Cancer Res. 2010, 70:2558-67) in the liver of the STAT3$^{C/C}$ mice. Accordingly, as suggested by the referee, we assessed P-STAT3 levels in the hearts of anti-IL6R treated mice and could show that they were significantly reduced, well correlating with the rescue from myocarditis (new Fig. 7F).

Other issues that require clarification:

1a) For many treatments discussed here survival is taken as endpoint. However, the bone-marrow chimera studies clearly suggest that the extent of myocarditis and survival does not correlate given that C>wt and wt>C mice both develop mild disease, but wt>C show the same reduced lifespan as naive Stat3C/C mice. How do authors know that the severely reduced life span results from myocarditis rather than systemic C3-dependent inflammation? Do C3 levels correlate with survival amongst different groups of bone marrow chimeras?

A/ We have now performed additional analysis of the bone marrow chimera experiments, including C>C chimeras as a control group in the new Fig. 6C-E and Supporting Fig. 3D-F, and assessing C3 deposition in all transplanted animals. We could show that WT->C chimeras develop a more aggressive myocarditis than that observed in the WT->WT and C->WT transplanted mice, similar to the C->C control mice. Surprisingly, despite similar death rates and age, the histopathological alteration observed in both the WT->C and the C->C groups was reduced with respect to non-transplanted Stat3$^{C/C}$ mice. This may be due to the early death after the transfer (between 12 and 15 days) that limited the accumulation and pathogenic action of the transplanted hematopoietic cells.

© EMBO

See Results section, end of page 8 and beginning of page 9). Moreover, C3 deposition could only be detected in $STAT3^{C/C}$ recipient mice, regardless of the genotype of the transplanted cells and correlating with premature death (new Fig. 6 F). This piece of data clearly shows that C3 production is contributed by non hematopoietic $STAT3^{C/C}$ cells, and that its accumulation in the heart may represent a crucial pathogenic factor in our model (Results section, page 9; Discussion section, page 12).

1b) This reviewer notes similar H&E, CD18 and picrosirius stains for antiCD4, wt>wt and wt>C mice although these groups show different survivals - why? What is the age of the mice from which histology is show in Figs 5E/G?

A/ We have shown better pictures of the H&E, CD18 and picrosirius red staining, including higher magnification photomicrographs and comparing survived and succumbed anti-CD4-treated mice (new Fig. 5 I). While surviving mice fail to show infiltration and fibrosis, succumbing mice display a high degree of both. The histology of both IgG-treated control and anti-CD4-treated surviving mice shown in new Fig. 5 I was performed on samples from mice reaching the experimental endpoint (60 days of age), while the histology of succumbing mice was necessarily performed at the age of death, between 30 and 40 days.

2) The Abstract/Discussion/Paper Explained sections give undue emphasis on a Th17 involvement when authors only show correlation with enhanced serum IL17 and enhanced in vitro Th17 polarization. Does IL17 depletion replicate the authors' observations with anti-IL6 antibodies?

A/ We have performed *in vivo* IL-17 neutralization and show the results in new Fig. 5F-G and new Supporting Information Fig S3A and B). Surprisingly, disease protection was much lower that that afforded by both CD4 depletion and IL-6R neutralization, suggesting a limited role for Th17 cells in our model (Results section, pages 7-8). This result is in agreement with recent data reporting limited role for Th17 cells in the inflammation phases of EAM (Baldeviano et al., Circulation Research 2010, 106:1646-55) and the absence of IL-17 producing cells in endomyocardial samples of patients with either acute myocarditis or DCM (Noutsias et al., European Journal of Heart Failure 2011, 13:611-618). We have accordingly modified the Abstract (page 2), Discussion (pages 11-12) and Paper explained sections (Results).

3a) Details referred to in the text can not readily be detected on some photomicrographs (i.e. in Fig 8 "lymphocytes" and the cell-types staining positive for P-Stat3).

A/ We have provided higher magnification photomicrographs from diseased patients in new Fig. 9A. However, we agree that cell type identification is not unequivocal and modified the text accordingly (page 10).

3b) Fig 5G should have an additional row documenting the phenotype of Stat3C/C mice, or even better C>C mice, for comparison - this magnification also makes it not possible to discern the cell types that stain for CD18. Please show high magnification.

A/ As suggested, we have included C>C chimeras as controls in new Fig. 6C-E and in new Supporting Fig. 3D-F (see also 1B, above), and shown higher magnification of the CD18 staining from this experiment (new Fig. 6B) as well as from the CD4 depletion experiment (new Fig. 5 I).

4) Why do the Western blots (Fig 4D) show simultaneously bands for Myh6 and MyBP-C3 when only blotted for one of these proteins in the 3rd and 4th panel?

A/ These are likely to be non-specific signals, as also suggested by a similar pattern in the representation of the Western blot analysis on rat and mouse heart extracts, shown in the datasheet of the MyBPC3 antibodies. We have included a comment to this end in the figure legend.

5) The representation of the Kaplan-Meier plots is confusing. Conventionally the position of a data point on the x-axis indicates the death of an animal thereby reducing the proportion of surviving animals to the next lower level as indicated by a vertical line. Accordingly the last data point is always above the axis. In Fig 7B the curves for the anti-IL6 treated animals confusingly remains at 100% after the first data point and this curve shows only 4 data points although n=7 in corresponding legend. From the data shown, it seems not valid to claim a significant difference between the IgG and anti-IL6 treated groups when the only difference is due to a single long surviving mouse in the anti-IL6 group.

A/ The plots were designed using the GraphPad software, where the Y axis shows the percentage of surviving mice at each time point, as indicated, rather than the number of succumbing mice. We have removed the dots and distinguished the groups by line colours in all Kaplan-Meier curves throughout the manuscript. The curve for the anti-IL6R-treated animals remaining at 100% after the first data point was a mistake and was corrected in new Fig. 8B. The raw data are as follows: out of a total of 7 treated mice, 5 survived up to day 60 and 2 succumbed, 1 at day 34 (corresponding to 85,7% survival) and 1 at day 35 (corresponding to 71,4% survival).

6a) The quantification of data in Figs 3A-C does not show a consecutive occurrence of CD4 accumulation (causally involved in mediating disease in Stat3C mice) and cx43 stain. Therefore authors should not make a causal connection between "inflammation" and "connexin 43 disorganization" in the Results section.

A/ It is true that CD4 accumulation and connexin 43 disruption start to occur at the same time, i.e. at three weeks of age, and thus that CD4 infiltration cannot be causally involved in mediating connexin 43 disorganization. However, at two weeks of age $Stat3^{C/C}$ mice already show accumulation of $Gr1^+$ cells, suggesting an initial inflammatory condition that is then exacerbated with age and that could well lead to tissue disorganization. We have clearly stated the $Gr1^+$-cx43 connection in the Results section, page 7.

6b) Similarly, authors refer in the Result sections to elevated IL6 level in humans with acute myocarditis; they should provide data for that for the samples the analyzed in Fig 8.

A/ We provide the measurement of circulating IL6 in human patients in the new Fig. 9 B, and show that indeed patients with acute myocarditis display significantly higher IL-6 levels than either healthy controls, patients with DCM or patients with chronic myocarditis.

Minor comments

1) All photomicrographs should have scale bars; referring to magnification is meaningless, because the size of the reproduced photomicrographs is different from the ones collected on the microscope.

A/ All photomicrographs were modified as requested.

2) Authors should state what Picrosirius red stains.

A/ The required information is indicated in the Methods (page 16) and Results section (pages 5 and 6).

3) In the first paragraph of the Discussion, authors refer to an incorrect phenotype (gp130Y757/Y757) for the mice used in the Hilfiker-Kleiner study; these mice were gp130Y757/flox.

A/ We thank the referee for spotting this mistake, that we have corrected. Indeed, it is an important difference since the gp130 floxed allele is only deleted in cardiomyocites in this model.

4) In Supporting Fig S2 N-values should be given, and the use of black and grey in the histograms in inconsistent with that in rest of manuscript (i.e. Fig 2, 6, 7)

A/ We apologyze for the inconsistency, and modified the figure legend accordingly. The *n* values are provided.

2nd Editorial Decision 20 January 2013

Please find enclosed the final reports on your manuscript. We are pleased to inform you that your manuscript is accepted for publication and is now being sent to our publisher to be included in the next available issue of EMBO Molecular Medicine.

I might be contacting you over the next few days to suggest some minor modifications to the text to improve impact.

Congratulations on your interesting work.

***** Reviewer's comments *****

Referee #1 (General Remarks):

The authors adressed all suggestions and concerns of ref. 1

Referee #2 (General Remarks):

None

Referee #3 (General Remarks):

The authors have addressed the extensive comments raised by the reviewers in a comprehensive and adequate manner and have included in the manuscript these suggestions and additional experiments.

I am satisfied with the revised version which now should be considered for publication in EMBO Mol Med
