# Supplementary material for: STAT3 activity is necessary and sufficient for the development of immune-mediated myocarditis in mice and promotes progression to dilated cardiomyopathy
Source: EMBO Mol Med. 2013 Mar 5;5(4):572–90. doi: 10.1002/emmm.201201876 (PMC3628107; doi:10.1002/emmm.201201876)
Supplement: Supplementary file 2 [file emmm0005-0572-sd2.pdf]

Table of Contents

Figure 1	STAT3 inhibition in EAM	pag.2
Figure 2	Levels of cytokines/chemokines mRNAs in the hearts of Stat3$^{C/C}$ mice	pag.3
Figure 3	IL-17 neutralization, CD4-depletion and Th1/Th2 cells differentiation	pag.4
Figure 4	Complement depletion and C3 deposition	pag.5
Figure 5	C3 levels upon IL-17 neutralization and CD4 depletion	pag.6
Table I	Infiltration degree in the tissues of Stat3$^{C/C}$ mice	pag.7
Table II	Cardiac ultrasound analysis of Stat3$^{C/C}$ and $^{WT/WT}$ mice	pag.8
Table III	Probes and oligonucleotide sequences used in Real time PCR	pag.9

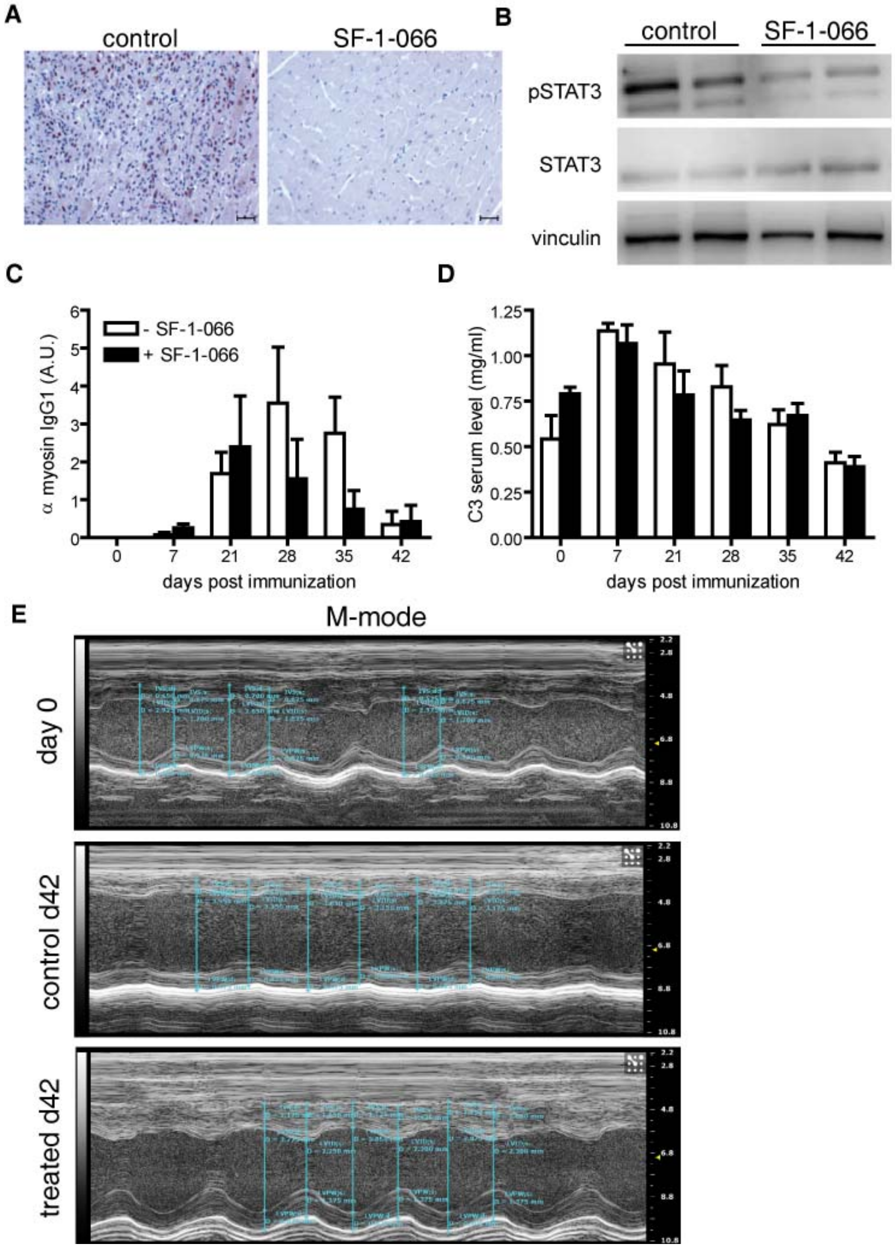

Supporting Information Figure 1. STAT3 inhibition in EAM.
EAM was induced in Balb/C mice with or without i.v. treatment with the STAT3 inhibitor SF-1-066, administered either from day 0 (mice sacrificed at day 21, **A, B**), or from day 21 (mice sacrificed at day 42, **C-E**). STAT3 phosphorylation was assessed at day 21 on heart samples by IHC (**A**), or by Western blotting on total heart extracts (**B**). Pictures are representative of two individual samples per condition. Scale bar: 50 µm. (**C, D**) The histograms show the mean±SEM of anti-myosin IgG1 (**C**) and C3 ELISAs (**D**) of mice at day 42 (*n*=4) either untreated (white bars) or treated (black bars) with SF-1-066 from day 21. (**E**) The indicated mice were analyzed by echocardiography at day 0 and day 42 post-immunization (see Fig. 1E-G). M-mode echocardiographic assessment of left ventricular diastolic function shows preserved contractile function in STAT3-inhibited mice at day 42.

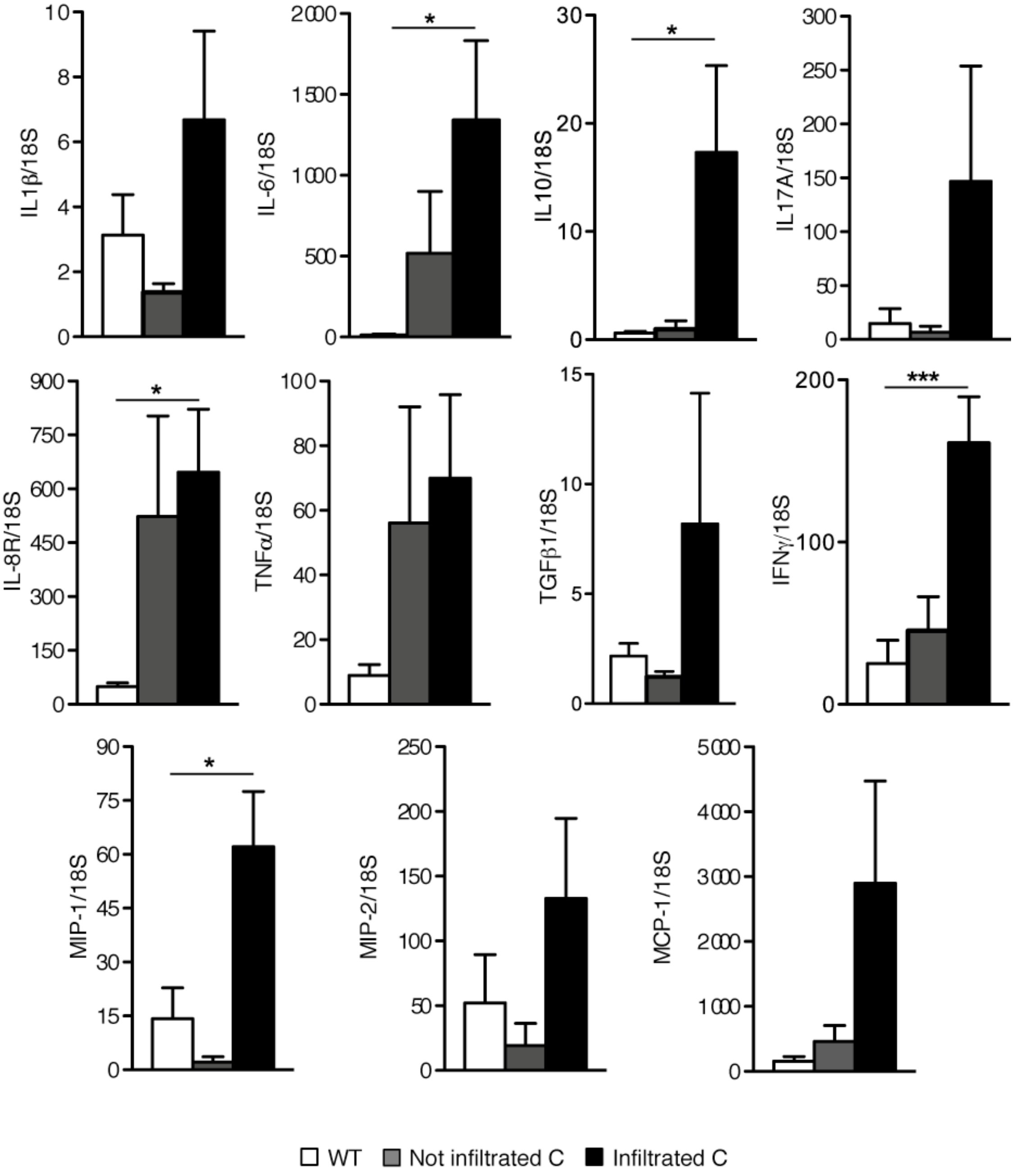

Supporting Information Figure 2. **High levels of proinflammatory cytokines/chemokines mRNAs in the hearts of infiltrated Stat3$^{C/C}$ mice.** Taqman RT-PCR analysis of the indicated cytokines/chemokines on total RNA from hearts of Stat3$^{WT/WT}$ (white bars, *n*=15), non-infiltrated (grey bars, *n*=5) and infiltrated (black bars, *n*=8) Stat3$^{C/C}$ mice. Bars represent mean±SEM of normalized levels. *, *P*=0.029 (IL-6); *P*=0.031 (IL-10); *P*=0.020 (IL8-R); *P*=0.0008 (IFNγ); *P*=0.020 (MIP-1).

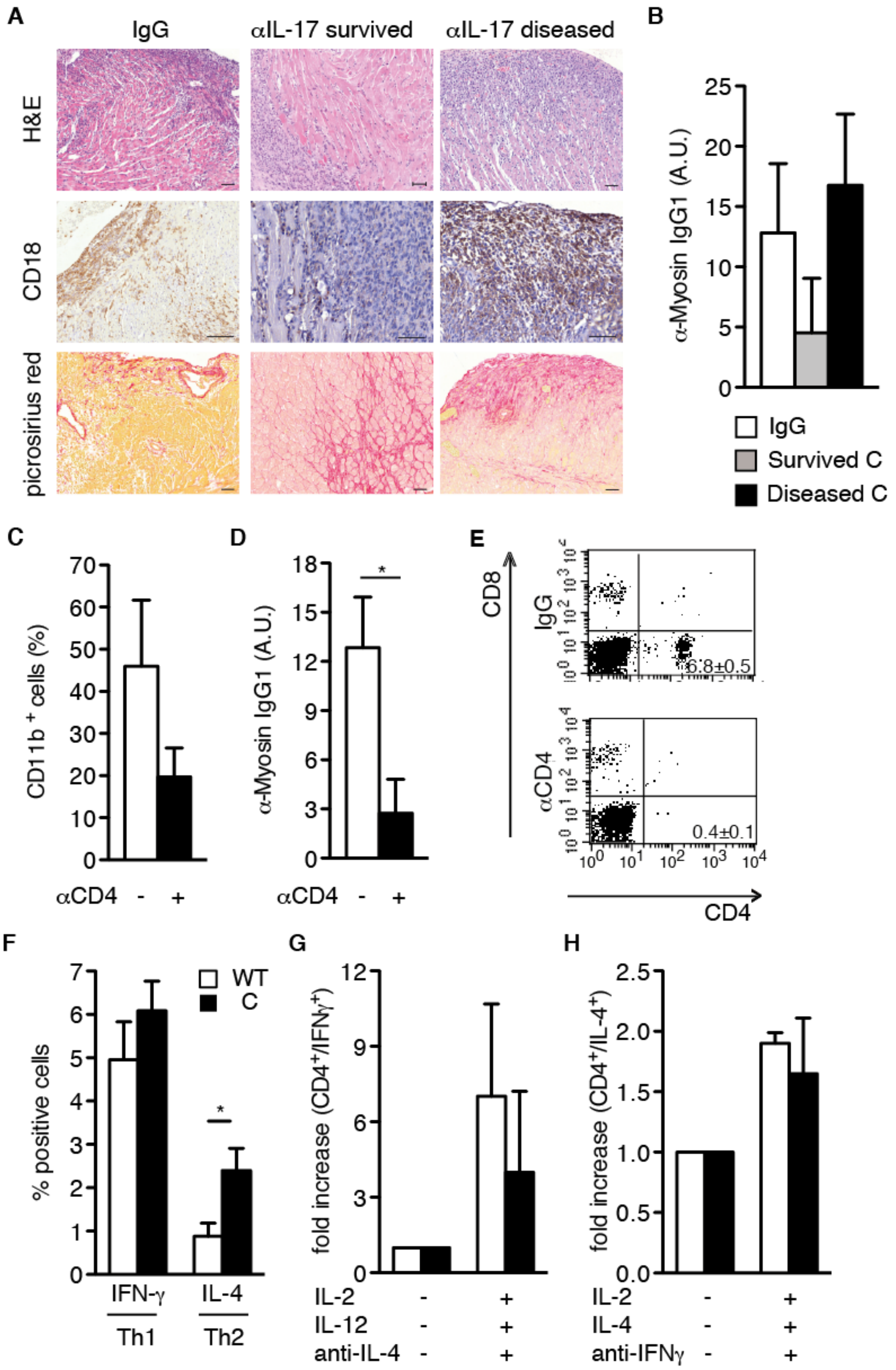

Supporting Information Figure 3. IL-17 neutralization, CD4-depletion and Th1/Th2 cells differentiation. (A) Analysis of STAT3$^{C/C}$ mice treated with anti-IL-17A mAbs or with IgGs as negative control. Scale bar: 50 µm. (**B**) Anti-myosin IgG1 ELISA on sera from the above mice at the indicated time points. IgGs-treated (white bars, *n*=10); anti-IL-17–treated (survived: grey bars, *n*=2; diseased, black bars, *n*=5). Heart infiltration by CD11b cells **(C)** and anti-α-myosin IgG1s **(D)** in Stat3$^{C/C}$ mice treated with anti-CD4 mAbs (*n*=4) or with IgGs as negative control (*n*=10). **P*=0.02. (**E**) CD4 depletion assessed by flow cytometry. (**F**) Cytofluorimetric analysis of IFNγ or IL-4 producing CD4^{+} T cells in the spleen of Stat3$^{WT/WT}$ (white bars, *n*=6) and Stat3$^{C/C}$ (black bars, *n*=6) mice. **P*=0.031. *In vitro* differentiation of Th1 **(G)** and Th2 **(H)** cells. Naïve CD4^{+} cells were purified from spleens of Stat3$^{WT/WT}$ (white bars, *n*=3) and Stat3$^{C/C}$ (black bars, *n*=4) mice and treated with the indicated cytokines.

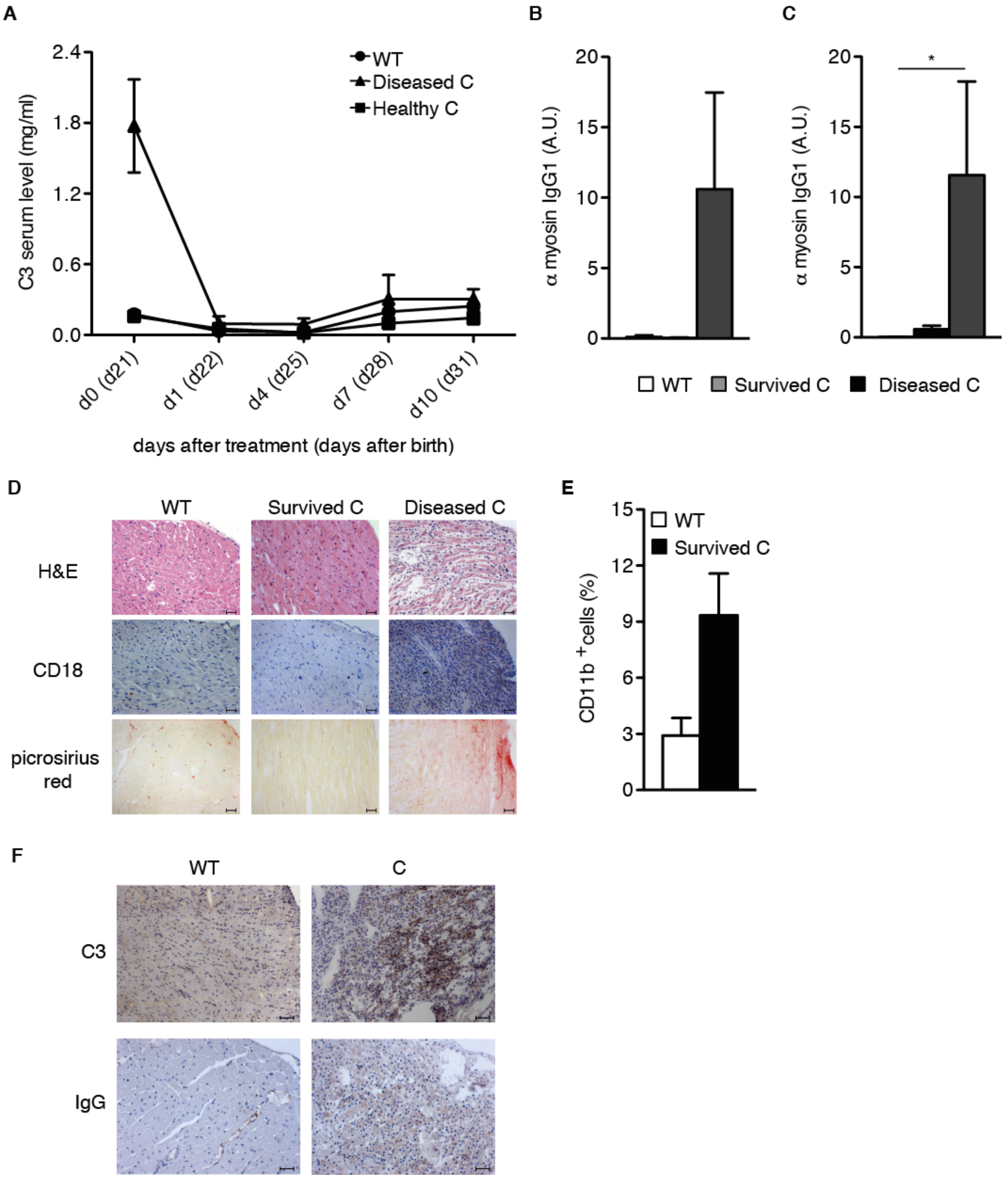

Supporting Information Figure 4. Complement depletion and C3 deposition. Stat3$^{C/C}$ or $^{WT/WT}$ mice were treated with CVF as described in the Methods section, starting at day 21 of age (d0 of treatment). C3 (**A**), myosin specific IgG1 at day 0 of treatment (**B**) or at time of sacrifice (**C**) were measured on sera of depleted mice of the indicated genotypes: Stat3$^{WT/WT}$ (circles, *n*=6) and Stat3$^{C/C}$ (healthy, squares, *n*=8; diseased, triangles, *n*=7). $^*P$=0.032. (**D**) IHC analysis of representative CVF-treated mice. (**E**) Flow cytometry of CD11b$^+$ cells in the hearts of CVF-treated Stat3$^{WT/WT}$ and surviving Stat3$^{C/C}$ mice. (**F**) Detection of C3 and IgG by IHC on the hearts of Stat3$^{WT/WT}$ and Stat3$^{C/C}$ mice. Pictures are representative of three to five samples analysed per genotype. (**D, F**). Scale bar: 50 μm.

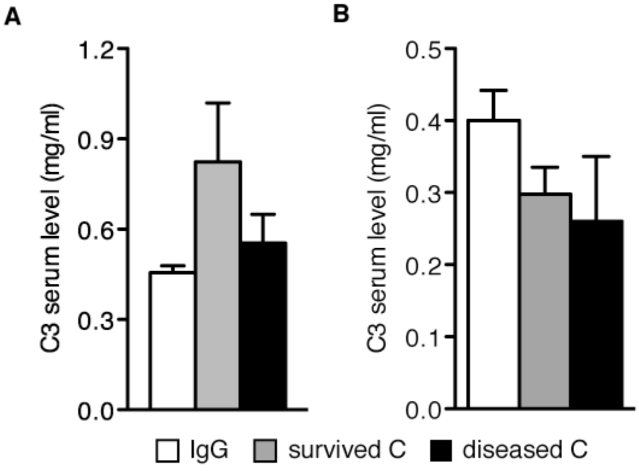

Supporting Information Figure 5. Neither IL-17 neutralization nor CD4 depletion down-regulate C3 levels in Stat3$^{C/C}$ mice. Circulating C3 levels were measured on sera from Stat3$^{C/C}$ mice treated with IgGs (white bar, *n*=4) and anti-IL-17A (survived mice, grey bar, *n*=2; diseased mice, black bar, *n*=5), or with IgGs (white bar, *n*=10) or anti-CD4 mAbs (survived, grey bar, *n*=4; diseased, black bar, *n*=2) (**B**).

	heart	sk. m.	spleen	LN	liver	lung	kidney	colon
1	++++	-	+	+	+	+	-	-
2	+++	-	+	+/-	+/-	-	-	-
3	++	-	+/-	-	-	-	-	-
4	++++	-	-	+/-	+/-	-	-	-

Supporting Information Table I. Infiltration degree in tissues of Stat3$^{C/C}$ mice. Four individual mice exhibiting different degrees of leukocyte infiltration in the heart were examined for infiltration of the indicated tissues by H&E analysis. Infiltration degrees were scored in blind by two independent operators.

	age	Stat3$^{WT/WT}$	Stat3$^{C/C}$	p-value
FS (%)	4 wks	48,1±2,7	40,2±2,2	0,045*
	> 4 wks	45,0±4,2	25,3±4,8	0,027*
LVEDD (mm)	4 wks	3,003±0,130	2,640±0,147	0,092
	> 4 wks	3,030±0,186	2,913±0,391	0,805

Supporting Information Table II. Cardiac ultrasound analysis of Stat3$^{C/C}$ and $^{WT/WT}$ mice. Mice of the indicated ages in weeks (wks) and genotypes were anesthetized by Zoletil 100 injected i.p. and subjected to echocardiography. FS, fractional shortening; LVEDD, left ventricular end-diastolic dimension. 4 wks: Stat3$^{WT/WT}$ mice, *n*=7; Stat3$^{C/C}$ mice, *n*=6;
> 4 wks (5-6 weeks old): Stat3$^{WT/WT}$ mice, *n*=6; Stat3$^{C/C}$ mice, *n*=3

probe	name	sequence
76	C3 left	5’-ACCTTACCTCGGCAAGTTTCT-3’
	C3 right	5’-TTGTAGAGCTGCTGGTCAGG-3’
26	IL1β left	5’-TTGACGGACCCCAAAAGAT-3’
	IL1β right	5’-GAAGCTGGATGCTCTCATCTG-3’
6	IL6 left	5’-GCTACCAAACTGGATATAATCAGGA-3’
	IL6 right	5’-CCAGGTAGCTATGGTACTCCAGAA-3’
41	IL10 left	5’-GCTCCTAGAGCTGCGGACT-3’
	IL10 right	5’-TGTTGTCCAGCTGGTCCTTT-3’
50	IL17A left	5’-TGTGAAGGTCAACCTCAAAGTC-3’
	IL17A right	5’-AGGGATATCTATCAGGGTCTTCATT-3’
19	IL23 left	5’-TCCCTACTAGGACTCAGCCAAC-3’
	IL23 right	5’-TGGGCATCTGTTGGGTCT-3’
69	IL8R left	5’-AAGGCCTTGAATGCTACGG-3’
	IL8R right	5’-AGGCATAGATGATGGGGTTAAG-3’
53	IL6R left	5’-ATCCTCTGGAACCCCACAC-3’
	IL6R right	5’-GAACTTTCGTACTGATCCTCGTG-3’
21	IFNγ left	5’-ATCTGGAGGAACTGGCAAAA-3’
	IFNγ right	5’-TTCAAGACTTCAAAGAGTCTGAGGTA-3’
69	MCP1 left	5’-AACTCTCACTGAAGCCAGCTCT-3’
	MCP1 right	5’-GTGGGGCGTTAACTGCAT-3’
66	MIP1 left	5’-AGATTCCACGCCAATTCATC-3’
	MIP1 right	5’-GCCGGTTTCTCTTAGTCAGGA-3’
66	MIP2 left	5’-AAAATCATCCAAAAGATACTGAACAA-3’
	MIP2 right	5’-CTTTGGTTCTTCCGTTGAGG-3’
101	RORγt left	5’-CACTGCCAGCTGTGTGCT-3’
	RORγt right	5’-TGCAAGGGATCACTTCAATTT-3’
72	TGFβ left	5’-TGGAGCAACATGTGGAACTC-3’
	TGFβ right	5’-CAGCAGCCGGTTACCAAG-3’
49	TNFα left	5’-TCTTCTCATTCCTGCTTGTGG-3’
	TNFα right	5’-GGTCTGGGCCATAGAACTGA-3’
sybergreen	Stat3C left	5’-CGTACCTGAAGACCAAGTTC-3’
	Stat3C right	5’-CTGCATTCTAGTTGTGGTTTGTCC-3’
sybergreen	Stat3wt left	5’-GCGGGCCATCCTAAGCACAAAG-3’
	Stat3wt right	5’-CACCTGCCGCAAATGTATTAACG-3’

Supporting Information Table III. Probes and oligonucleotide sequences used in Real time PCR.
